# Identification of kinase modulators as host-directed therapeutics against intracellular methicillin-resistant *Staphylococcus aureus*

**DOI:** 10.3389/fcimb.2024.1367938

**Published:** 2024-03-25

**Authors:** Robin H. G. A. van den Biggelaar, Kimberley V. Walburg, Susan J. F. van den Eeden, Cassandra L. R. van Doorn, Eugenia Meiler, Alex S. de Ries, Annemarie H. Meijer, Tom H. M. Ottenhoff, Anno Saris

**Affiliations:** ^1^ Leiden University Center for Infectious Diseases, Leiden University Medical Center, Leiden, Netherlands; ^2^ Institute of Biology Leiden, Leiden University, Leiden, Netherlands; ^3^ Global Health Medicines R&D, GlaxoSmithKline, Tres Cantos, Spain

**Keywords:** methicillin-resistant *Staphylococcus aureus*, host-directed therapy, intracellular infection, published kinase inhibitor set (PKIS), EGFR/HER kinase family, AMPK, autophagy, zebrafish embryo

## Abstract

The increasing prevalence of antimicrobial-resistant *Staphylococcus aureus* strains, especially methicillin-resistant *S. aureus* (MRSA), poses a threat to successful antibiotic treatment. Unsuccessful attempts to develop a vaccine and rising resistance to last-resort antibiotics urge the need for alternative treatments. Host-directed therapy (HDT) targeting critical intracellular stages of *S. aureus* emerges as a promising alternative, potentially acting synergistically with antibiotics and reducing the risk of *de novo* drug resistance. We assessed 201 ATP-competitive kinase inhibitors from Published Kinase Inhibitor Sets (PKIS1 and PKIS2) against intracellular MRSA. Seventeen hit compounds were identified, of which the two most effective and well-tolerated hit compounds (i.e., GW633459A and GW296115X) were selected for further analysis. The compounds did not affect planktonic bacterial cultures, while they were active in a range of human cell lines of cervical, skin, lung, breast and monocyte origin, confirming their host-directed mechanisms. GW633459A, structurally related to lapatinib, exhibited an HDT effect on intracellular MRSA independently of its known human epidermal growth factor receptor (EGFR)/(HER) kinase family targets. GW296115X activated adenosine monophosphate-activated protein kinase (AMPK), thereby enhancing bacterial degradation via autophagy. Finally, GW296115X not only reduced MRSA growth in human cells but also improved the survival rates of MRSA-infected zebrafish embryos, highlighting its potential as HDT.

## Introduction

Antibiotics are essential to treat bacterial infections, yet their effectiveness is increasingly threatened by antimicrobial resistance (AMR). In 2019, AMR caused approximately 1.27 million deaths, a number projected to reach ten million by 2050 (O’Neill, 2016; Murray et al., 2022). *S. aureus* is the second deadliest bacterial species associated with AMR, and its methicillin-resistant form (MRSA) claims over 100,000 lives every year (Cassini et al., 2019; Murray et al., 2022). While *S. aureus* is commonly found as a commensal in the upper respiratory tract and on the skin of around 1/5 of healthy individuals, it may turn into an opportunistic pathogen, causing severe infections in immunocompromised individuals, such as soft tissue infections, osteomyelitis, endocarditis, pneumonia and sepsis (Wertheim et al., 2005; Huitema et al., 2021). Recently, intracellular life stages of *S. aureus* have been recognized to play a critical role in the pathogenesis, supporting escape of immune responses and antibiotic treatment (Prajsnar et al., 2012; Lehar et al., 2015; Prajsnar et al., 2021; Rodrigues Lopes et al., 2022; Goormaghtigh and Van Bambeke, 2024). Efforts to develop an effective vaccine against *S. aureus* have been unsuccessful so far. Treatment of MRSA depends on last-resort antibiotics including vancomycin, linezolid and daptomycin; however, vancomycin resistance is already on the rise (Shariati et al., 2020). One possible alternative strategy to treat *S. aureus* infection is host-directed therapy, in which infected host cells are modulated to create an inhospitable environment for invading bacteria (Bravo-Santano et al., 2019b; Bravo-Santano et al., 2019c).

*S. aureus* invades and survives inside phagocytes as well as non-phagocytic cells (including epithelial cells, endothelial cells, fibroblasts, keratinocytes and osteoblasts) (Prajsnar et al., 2012; Lehar et al., 2015; Prajsnar et al., 2021; Rodrigues Lopes et al., 2022; Goormaghtigh and Van Bambeke, 2024). *S. aureus* actively invades non-phagocytic cells by binding to fibronectin receptors to stimulate their internalization (Sinha et al., 1999; Rodrigues Lopes et al., 2022). Subsequently, *S. aureus* can temporarily change into a small colony variant phenotype to withstand lysosomal degradation, which reverts to the more virulent phenotype in response to favorable environmental conditions (Tuchscherr et al., 2011; Leimer et al., 2016; Rollin et al., 2017). In addition, *S. aureus* escapes from phagosomes to the cytosol by secreting amphipathic peptides and pore-forming toxins (Giese et al., 2011; Chi et al., 2014; Grosz et al., 2014). Cytosolic bacteria can be recaptured by the autophagy pathway, but whether this mechanism is beneficial for the host or *S. aureus* is still under debate (Schnaith et al., 2007; Bravo-Santano et al., 2018; Prajsnar et al., 2021). HDTs may be used to overcome the immune evasion strategies of intracellular *S. aureus*, in particular in conjunction with standard-of-care antibiotics that show limited activity intracellularly (Cristina and Françoise, 2003; Maritza et al., 2006). Furthermore, HDTs are expected to be active against antibiotic-resistant strains of *S. aureus* and the risk of *de novo* resistance may be reduced because there is no direct selection pressure on the bacteria.

Recent studies have identified potential HDTs to treat *S. aureus*, including caspase inhibitor Q-VD-OPH, pyrimidine synthesis inhibitor *N*-phosphonacetyl-L-aspartate, anti-inflammatory agents from herbal medicine, and the synthetic host-defense peptide DRGN-1 (Chung et al., 2017; Jatana et al., 2018; Li et al., 2020; Shi et al., 2020; Alphonse. et al., 2021). Furthermore, statins were identified as potential HDTs, but also acted as antibiotics at high concentrations (Horn et al., 2008; Graziano et al., 2015). Finally, kinase inhibitors Ibrutinib, Dasatinib and Crizotinib were recently identified as potential HDTs against intracellular MRSA, showing that small molecule inhibitors targeting host kinases might be effective (Bravo-Santano et al., 2019b). To further explore this area, we screened compounds from the Published Kinase Inhibitor Sets (PKIS) 1 and 2 for their efficacy in cell-based infection models, followed by *in vivo* safety and efficacy tests in zebrafish embryos (Elkins et al., 2016; Drewry et al., 2017). This screen resulted in two hit compounds mediating HDT effects on MRSA through different kinase families.

## Materials and methods

### Reagents

Gentamicin sulfate, dimethyl sulfoxide (DMSO), Triton X-100 (used at 1%), mTOR inhibitor rapamycin (used at 1 μM) and bafilomycin A1 (used at 100 nM) were all purchased from Sigma-Aldrich (Merck, Darmstadt, Germany). Sapitinib (AZD8931) and lapatinib were ordered from Selleck Chemicals (Houston, TX, USA) and SignalChem (Richmond, BC, Canada), respectively. Kinase inhibitors from the published kinase inhibitor sets (PKIS)1 and PKIS2 were kindly provided by GlaxoSmithKline Global Health Medicines R&D (GSK) and the Structural Genomics Consortium of the University of North Carolina at Chapel Hill (SGC-UNC) (Elkins et al., 2016; Drewry et al., 2017). Larger quantities of GW296115X were obtained from AOBIOUS (Gloucester, MA, USA). All PKIS compounds were dissolved in DMSO at 10 mM. Adenosine monophosphate-activated protein kinase (AMPK) activator compound 991 (ex229) (Xiao et al., 2013) (Spirochem, Basel, Switzerland; used at 25 μM) was kindly shared by Eline Brombacher and Dr. Bart Everts (Leiden university Medical Center, Leiden, Netherlands). Chemical structures were generated from published simplified molecular-input line-entry specification (SMILES) using ChemDraw (PerkinElmer, Waltham, MA, USA).

### Cell culture

Both HeLa cervical adenocarcinoma cells and MelJuSo melanocytes were cultured in Iscove’s Modified Dulbecco’s Medium (IMDM), A549 lung carcinoma cells were cultured in Dulbecco’s Modified Eagle Medium (DMEM), and both MCF7 mammary gland adenocarcinoma cells and THP-1 acute monocytic leukemia cells were maintained in Dutch-modified Roswell Park Memorial Institute (RPMI) 1640 medium (all from Gibco, ThermoFisher Scientific, Waltham, MA, USA) at 37°C/5% CO_2_. Media were supplemented with 10% fetal bovine serum (FBS; Greiner Bio-One, Alphen a/d Rijn, the Netherlands) and 100 units/ml penicillin and 100 µg/ml streptomycin (Gibco). Before experiments, THP-1 cells were differentiated to adherent macrophage-like cells using 5 ng/ml phorbol 12-myristate 13-acetate (PMA) (InvivoGen, San Diego, CA, USA) for 24 h, followed by a recovery period of 48-72 h.

### Bacterial culture

*S. aureus* strains, listed in **Table 1**, were recovered from a frozen glycerol stock and cultured in Difco Tryptic Soy (TS) broth (BD Biosciences, Franklin Lakes, NJ, USA) overnight at 37°C. To retain the plasmids of fluorescent or bioluminescent bacteria, the TS broth was supplemented with tetracycline or chloramphenicol (both from Sigma Aldrich) to select for bacteria that harbour the plasmid containing resistance genes. MRSA was subcultured 1:33 two to three hours prior to experiments to obtain a log-phase bacterial culture.

**Table 1:** *S. aureus* strains used in this study.

Strain	MRSA	Plasmid	Antibiotic for selection	Reference
USA300 LAC JE2	yes	pMV158-GFP	5 µg/ml tetracycline	(Nieto and Espinosa, 2003)
USA300 LAC JE2	yes	pRP1195-luxABCDE	10 µg/ml chloramphenicol	(Plaut et al., 2013)
USA300 BK 11540	yes	–	–	(Battouli et al., 2005)
LUH 15392	no	–	–	–
LUH 15393	no	–	–	–

### Gentamicin protection assay

The cells were resuspended in cell culture medium without antibiotics, seeded with 10,000 cells/well into Costar flat-bottom 96-well plates (Corning, Amsterdam, the Netherlands), and were incubated overnight at 37°C/5% CO_2_. The next day, the bacteria were centrifuged at ≥1800 *g* for 15 min and resuspended in cold PBS + 5 mM ethylenediaminetetraacetic acid (EDTA). The number of CFUs present in the bacterial suspension was estimated from the optical density at 600 nm (OD600) by assuming 8x10^8^ CFUs to be present in a bacterial suspension with OD600 = 1, based on prior titrations. The bacterial suspension was diluted in cell culture medium without antibiotics and added to the cells. Accuracy of the multiplicity of infection (MOI) was validated by plating serial dilutions of the MRSA inoculums on Difco TS agar plates (BD Biosciences). The calculated mean MOI throughout all experiments was 6.8 ± 4.6 (mean ± SD). Infected cells were centrifuged for 3 min at 150 g and incubated for 1 hour at 37°C/5% CO_2_. Next, the cells were incubated with fresh cell culture medium supplemented with 30 µg/ml gentamicin sulphate (Lonza BioWhittaker, Basel, Switzerland) for 15 min at 37°C/5% CO_2_ to kill residual extracellular bacteria. Infected cells were incubated overnight at 37°C/5% CO_2_ in cell culture medium in the presence of kinase inhibitors and 5 µg/ml gentamicin sulphate-containing medium to prevent the growth of extracellular bacteria. The solvent of the compounds, DMSO, was used at equal % v/v as a negative control. For the screen, each compound was tested in triplicate, on separate cell culture plates, resulting in triplicate values for each kinase inhibitor.

### Compound screen by flow cytometry

HeLa cells were harvested by trypsinization and fixed with 1% paraformaldehyde. Samples were measured on a FACSLyric with HTS extension (BD Biosciences). Flow cytometry data were analysed using FlowJo version 10 (TreeStar, Ashland, OR, USA). HeLa cells positive for GFP were gated to calculate the percentage of MRSA-infected cells. The cell count was used as surrogate readout of cell viability.

To identify potential “hit” compounds, the flow cytometry data were used to calculate standard z-scores using z = (x-μ)/σ – z_neg_, where x is total event count or the percent of GFP-positive cells from a single well, μ is the mean from wells of the plate, σ is the standard deviation of the plate and z_neg_ is the mean z-score of the negative control of the plate (i.e., DMSO) (Korbee et al., 2018). To minimize the possibility that the presence of extreme values on a plate affected the z-scores, raw values that deviated two-fold from the negative controls were excluded when calculating plate means and standard deviations as used in the aforementioned formula. Compounds were considered a “hit” when z-score_MRSA+_ ≤ -2 and z-score_HeLa_ ≥ -3 for cell count. In cases where compounds were screened twice, the average values were used to select hit compounds.

### Counting colony-forming units

Infected cells were lysed in sterile water + 0.05% UltraPure SDS solution (Invitrogen, ThermoFisher Scientific) to release intracellular bacteria. Bacterial suspensions and lysates of cells infected with MRSA were 5-fold serially diluted and 10-μl drops were plated on square TS agar plates. The plates were left to dry and incubated overnight at 37°C/5% CO_2_.

### Bacterial growth assay

MRSA cultures at a concentration corresponding to an absorbance of 0.1 at 600 nm were incubated with the kinase inhibitors at 10 μM in flat-bottom 96-well plates. The absorbance was measured using an EnVision plate reader (PerkinElmer). The plates were incubated at 37°C overnight and the absorbance was measured again the next day. Real-time measurement of bioluminescence by the MRSA-lux strain was performed in a white flat-bottom 96-wells plate using a SpectraMax i3x plate reader and an integration time set at 9000 ms.

### Lactate dehydrogenase-release cytotoxicity assay

Before harvesting or lysing the cells of the intracellular infection assays, the supernatant from the cells was collected to quantify LDH release by the cells using the LDH cytotoxicity detection kit (Roche, Merck, Darmstadt, Germany) according to the manufacturer’s instructions. Triton X-100 results in maximum LDH release by permeabilizing the cells and was used as positive control. Quantification was performed with the EnVision plate reader.

### Kinase inhibition data and KinMap analysis

Kinase inhibition profiles of the PKIS compounds at 1 µM were previously determined and are publicly available (Elkins et al., 2016; Drewry et al., 2017). Of note, kinase inhibition profiles of PKIS1 compounds were determined at 1 µM against 224 kinases by Caliper assay (Caliper), whereas kinase inhibition profiles of PKIS2 compounds were determined against 406 kinases by KinomeScan (DiscoverX). The KinMap phylogenetic tree was constructed using kinase inhibition data of 11 PKIS1 hit compounds versus 198 kinases and 9 PKIS2 hit compounds versus 393 kinases, including 2 hit compounds were part of both PKIS1 and PKIS2 (Eid et al., 2017). Kinase inhibition data for mutant forms of kinases, phosphatidylinositol kinases and sphingosine kinases were not included.

### Predicting cationic amphiphilic drugs

Identification of cationic amphiphilic drugs (CADs) was performed in line with a previous publication (Muehlbacher et al., 2012; Tummino et al., 2021). First, a prediction was performed by determining the calculated pK_a_ values (with K_a_ being the acid dissociation constant) and LogP values (with P being the octanol:water coefficient). Compounds with pKa ≥ 7.4 and logP ≥ 3 were considered potential cationic amphiphilic drugs.

### NBD-PE incorporation assay

To confirm CAD prediction (Tummino et al., 2021), HeLa cells were incubated with compounds at 10 µM and nirobenzoxadiazole-labelled 16:0 phosphatidylethanolamine (NBD-PE) tracer (Invitrogen) at 50 µM overnight at 37°C. The next day, the tracer was removed by washing the cells three times in PBS containing 100 mg/l calcium chloride and 100 mg/l magnesium chloride. NBD-PE incorporation was determined using a SpectraMax i3x plate reader at excitation wavelength 470/15 nm and emission wavelength 545/25 nm.

### Protein expression analysis by flow cytometry

To determine the expression of the HER kinase family, cells were harvested using PBS + 5 mM EDTA and fixed in 1% paraformaldehyde for 20 min at RT. To determine phosphorylation of ACC1 at Ser80, cells were harvested by incubation in pre-warmed trypsin-EDTA, which was neutralized after 5 min with an equal volume of FBS, and fixed in 4% ultra-pure formaldehyde (Bio Trend, Cologne, Germany) for 20 min at RT. For intracellular staining, the cells were permeabilized in ice-cold methanol for 20 min. Subsequently, the cells were blocked in PBS + 0.5% bovine serum albumin + 0.005% NaN_3_ (both from Sigma-Aldrich; FACS buffer) + 5% human serum for 5 min. The cells were labelled with primary and secondary antibodies (listed in **Supplementary Table S1**) in FACS buffer. Samples were measured on the FACSLyric and analysed using FlowJo 10 (FlowJo LCC, Ashland, OR, USA).

### Protein expression analysis by western blot

HeLa cells were seeded with 100,000 cells/well in 24-wells plates. In short, the cells were lysed in EBSB buffer (10% v/v glycerol, 3% SDS, 100 mM Tris-HCl, pH 6.8) supplemented with one tablet of cOmplete EDTA-free protease inhibitor cocktail (Sigma-Aldrich) and proteins were denaturated at 95°C for 5 min. Protein concentrations were determined using a Pierce™ BCA protein assay kit (Thermo Fisher Scientific). Samples were diluted in Laemmli sample buffer and loaded on a 15-well 4–20% Mini-PROTEAN^®^ TGX™ Precast Protein Gel for electrophoresis (both from Bio-Rad, Hercules, CA, USA). Samples were transferred to methanol-activated Immun-Blot PVDF membranes (Biorad) in Tris-glycine buffer (25 mM Tris, 192 mM glycine and 20% methanol). Membranes were blocked for 1h in PBS supplemented with 5% w/v non-fat dry milk. Next, the membranes were cut and each protein was separately stained with primary antibodies against actin and LC3B in PBS supplemented with 5% w/v non-fat dry milk, or against AMPKα and AMPKα-pThr172 in PBS supplemented with 5% BSA (antibodies listed in **Supplementary Table S1**). Membranes were stained with secondary antibodies in PBS supplemented with 5% w/v non-fat dry milk. After each staining, membranes were washed four times in PBS + 0.1% Tween20. Prior to revelation, the membranes were incubated for 10 min in enhanced chemiluminescence (ECL) Prime Western Blotting System reagent (GE Healthcare, Hoevelaken, The Netherlands). Imaging was performed on an iBright Imaging System (Invitrogen, Breda, The Netherlands). Protein bands were quantified using Fiji software (Schindelin et al., 2012).

### Zebrafish embryo toxicity test

Zebrafish were handled in compliance with local animal welfare regulations as overseen by the Animal Welfare Body of Leiden University (License number: 10612) and maintained according to standard protocols (http://zfin.org). All protocols adhered to the international guidelines specified by the EU Animal Protection Directive 2010/63/EU. All experiments with zebrafish were done on embryos or larvae up to 5 days post fertilization, which have not yet reached the free-feeding stage and which were of the wild type AB/TL line. Fertilized embryos were maintained at 28°C and kept in egg water containing 60 μg/ml Instant Ocean Sea Salt (Sera, Heinsberg Germany). Zebrafish embryos were dechorionated with Dumont #5 forceps (Fine Science Tools Inc., Foster City, CA, USA) at 24 hours post fertilization (hpf) and transferred to 96-wells plates with each well containing a single embryo. Subsequently, the embryos were treated with kinase inhibitors or DMSO at 30 hpf. The health status of the embryos was assessed at 120 hpf. Healthy embryos were given a health score of 5. Health points were withdrawn for each of the following symptoms: no responsiveness to physical stimulation, cranial malformations, oedema or tail malformations. Dead embryos were given a health score of 0.

### Zebrafish embryo efficacy test

Zebrafish embryos were dechorionated at 24 hpf. The OD600 of a log-phase MRSA culture was centrifuged and dissolved in PBS with 2% polyvinylpyrrolidone (Merck) to improve homogeneity of the bacterial suspension. The OD600 was measured and the bacterial suspension was diluted to a final concentration of 10^9^ CFUs/ml accordingly. The preparation of injection needles and the equipment used for microinjections have been previously described (Benard et al., 2012). At 28 hpf, zebrafish embryos were anesthetized in 200 µg/ml buffered ethyl 3-aminobenzoic acid (Tricaine, Sigma-Aldrich) and injected in the blood island with a bacterial dose of 1 nl containing 1000 CFUs of MRSA. Infected embryos were collected and randomly distributed over 6-wells plates with ten embryos/well. At 30 hpf, the infected embryos were given kinase inhibitors or DMSO as negative control. Survival of the embryos was scored at 48, 72, 96 and 120 hpf.

### Statistical analysis

Statistical analysis was performed using GraphPad Prism 9 software (GraphPad Software, CA, USA). Kinase inhibition data were compared between the selected 18 PKIS hit compounds and the other PKIS compounds and tested for statistical significance of observed differences using Mann-Whitney tests. Data obtained from the CFU assay and LDH assay were tested for significant differences between groups by performing Friedman tests for matched samples and Dunn’s multiple comparisons tests for *post-hoc* analysis. Data obtained with the NBD-PE incorporation assay were tested for significant differences between groups using a one-way ANOVA and Dunnett’s multiple comparisons test. Zebrafish toxicity data were assessed for significant differences between unmatched compound-treated and untreated groups using Kruskal-Wallis tests and Dunn’s multiple comparisons tests for *post-hoc* analysis. Zebrafish embryo survival data were compared between compound-treated and DMSO-treated embryos using Kaplan-Meijer survival analysis. Differences between groups resulting in *p*-values < 0.05 were considered statistically significant.

## Results

### Screening 201 kinase inhibitors from the PKIS library identifies 17 compounds as active against intracellular MRSA

In order to identify new HDTs, we set up an intracellular infection model for MRSA using a GFP-expressing strain (**Supplementary Figure S1A**). HeLa cells were actively invaded by the bacteria and resulted in sufficient infected cells to screen for HDTs (**Supplementary Figure S1B**). Over time, the percentage of infected cells slowly declines due to a combination of host cells that clear the bacteria and lysis of infected host cells with replicating bacteria (**Supplementary Figures S1B-E**). A low concentration of 5 µg/ml gentamicin was added to the cell culture medium to restrict the bacteria from replicating extracellularly upon lysis of host cells (**Supplementary Figures S1E, F**).

**Figure 1:**
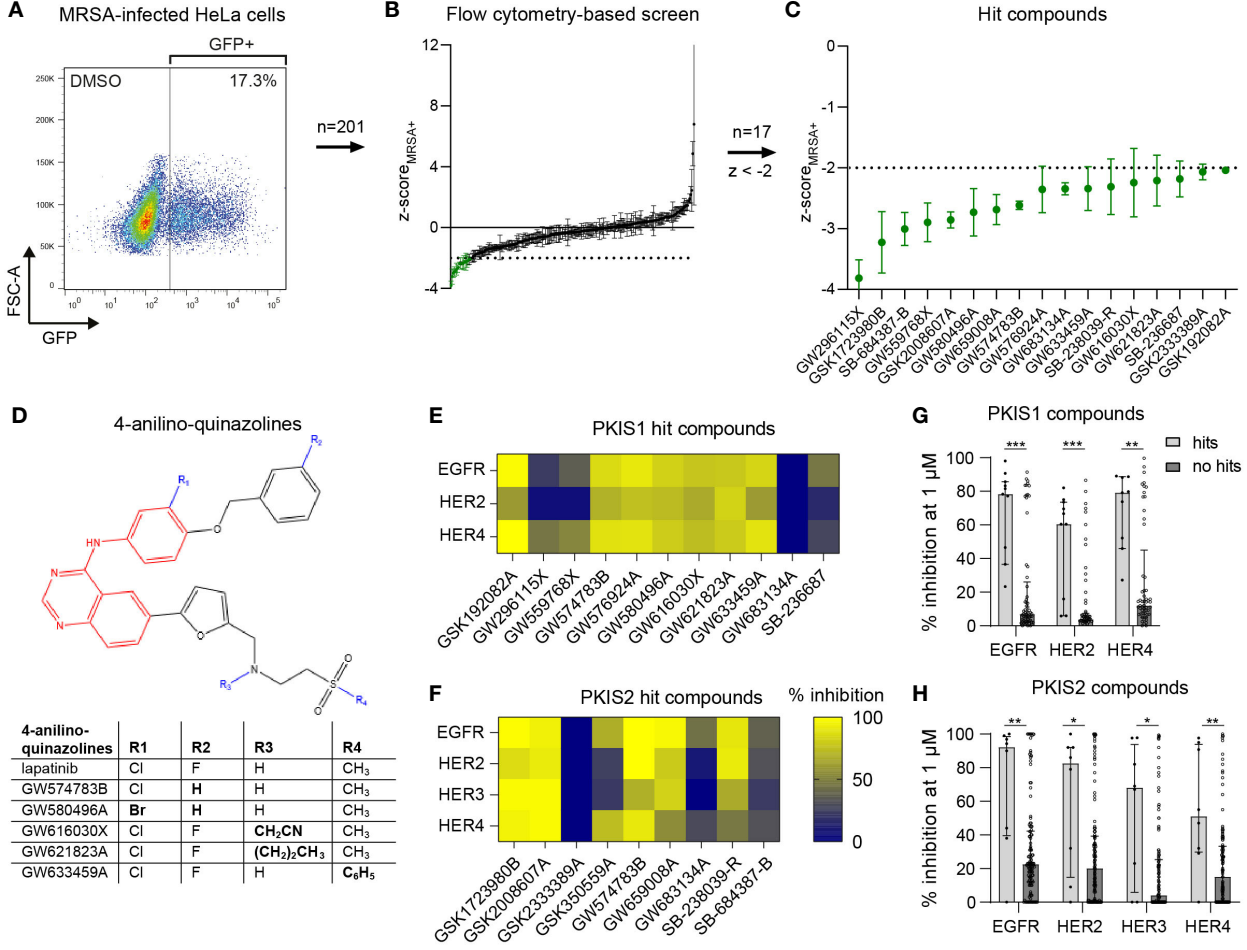
Identification of PKIS compounds inhibiting intracellular growth of MRSA. **(A)** GFP-positive MRSA-infected HeLa cells were gated to determine the percentage of infected cells. **(B)** The DMSO control and 201 PKIS compounds were screened to assess their effect on MRSA bacterial burden, expressed as average z-scores of GFP-positive cells (z-score_MRSA+_). **(C)** The 17 hit compounds with z-score_MRSA+_ < -2 are shown. The screen was performed with three replicates for the PKIS compounds. Error bars represent standard deviations. **(D)** Structural resemblance of 4-anilino-quinazoline hit compounds with lapatinib. **(E, F)** The capacity of both PKIS1 **(E)** and PKIS2 **(F)** hit compounds to inhibit the HER kinase family is shown. Kinase inhibition data of HER3 were only available for PKIS2 compounds. **(G, H)** A comparison is made between hit compounds and non-hit PKIS compounds in their capacity to inhibit the human epidermal growth factor receptor (HER) kinase family. Bars and error bars show the median ± interquartile range. Each datapoint represents a PKIS compound. Statistical significance of observed differences was tested using Mann-Whitney tests (*p < 0.05; **p < 0.01; ***p < 0.001).

We selected 201 PKIS inhibitors that previously were found non-cytotoxic and showed an effect in cell lines infected with *Salmonella enterica* serovar Typhimurium and *Mycobacterium tuberculosis* (van den Biggelaar et al., 2023). The compounds were screened at 10 µM for their capacity to reduce the bacterial burden of HeLa cells infected with GFP-expressing MRSA. After 24 h the cells were harvested and analyzed by flow cytometry (**Figure 1A**). The screen resulted in 20 compounds that reduced the MRSA intracellular burden below the efficacy threshold of z-score_MRSA+_ < -2, three of which were excluded from further analysis due to inconsistent results between replicates (**Figures 1B, C**; **Supplementary Table S2**). Compound GW296115X showed the strongest inhibition of intracellular MRSA with z=-3.82, representing a reduction from 14.6% ± 1.0% (mean ± SD) to 2.1% ± 0.26% MRSA-infected cells. None of the hit compounds resulted in cell viability levels below the threshold used for exclusion (z-score_HeLa_ < -3; **Supplementary Table S2**).

Several hit compounds belonged to the same chemotype. Six compounds, namely GW574783B, GW576924A, GW580496A, GW616030X, GW621823A, and GW633459A, are 4-anilino-quinazolines that bear a strong structural resemblance to lapatinib, used to treat breast cancer by targeting the human epidermal growth factor receptor (EGFR)/(HER) family (**Supplementary Table S3**; **Figure 1D**) (Lackey, 2006; Petrov et al., 2006; Qiu et al., 2008). Published kinase inhibition profiles of the hit compounds suggested the EGFR/HER kinase family as being the most likely common host target involved in inhibition of intracellular MRSA (**Figures 1E, F**) (Elkins et al., 2016). Moreover, the hit compounds inhibited the HER kinase family significantly more than other compounds from the PKIS library (**Figures 1G, H**). Another dominant chemotype among the hit compounds was formed by 4-pyrimidinyl-ortho-aryl-azoles, including GSK1723980B, GSK2008607A, SB-238039-R and SB-236687 (**Supplementary Table S3**; **Supplementary Figure S2**) (Elkins et al., 2016; Drewry et al., 2017). These compounds display highly promiscuous kinase inhibition profiles, making it difficult to identify common kinase targets. The seven remaining hit compounds belong to a diversity of chemotypes without structural relationships.

### Compounds GW633459A and GW296115X significantly reduced the bacterial burden in different cell types through a host-directed mechanism without compromising host cell viability

**Figure 2:**
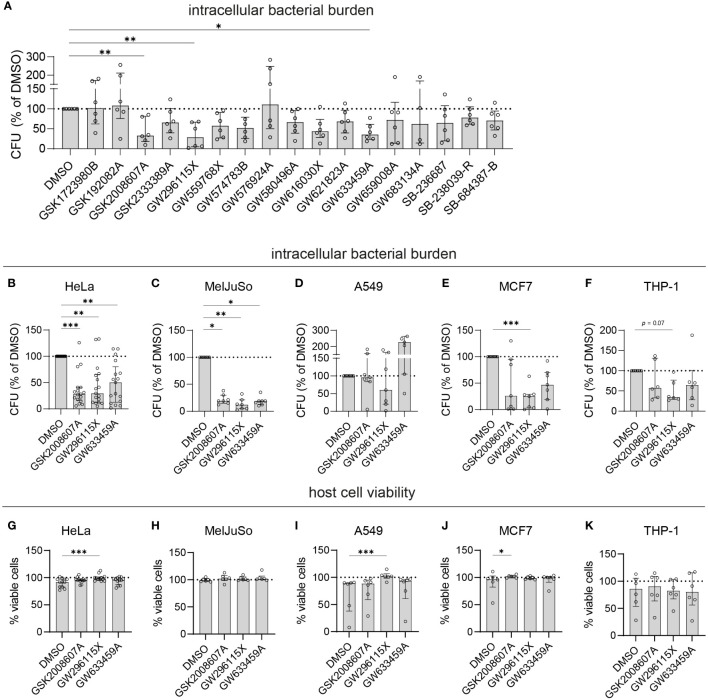
Validation of selected PKIS hit compounds. **(A)** The efficacy of the PKIS hit compounds against intracellular MRSA was determined by CFU count and given as the percentage of the DMSO control. **(B–F)** The efficacy of GSK2008607A, GW296115X and GW633459 on the bacterial burden of MRSA-infected HeLa cells (B, n=17), MelJuSo cells (C, n=7), A549 cells (D, n=7), MCF7 cells (E, n=7) and PMA-differentiated THP-1 cells (F, n=6) was determined by CFU count. **(G–K)** The effect of the compounds on cell viability was assessed using LDH-release assay for HeLa cells **(G)**, MelJuSo cells **(H)**, A549 cells **(I)**, MCF7 cells **(J)** and PMA-differentiated THP-1 cells **(K)**. Bars and error bars show the median ± interquartile range. Each datapoint represents the results of an independent experiment. Statistical significance of observed differences was tested in comparison to the DMSO controls using Friedman tests (*p < 0.05; **p < 0.01; ***p < 0.001).

To select the most effective and robust hit compounds, validation experiments were conducted. The compounds were added to planktonic MRSA cultures at 10 µM to assess their potential direct antimicrobial effects. Except GW659008A, which inhibited planktonic MRSA growth by 67.3% ± 24.9%, the other hit compounds did not affect bacterial growth (< 20% inhibition), indicating that they act in a host-directed manner (**Supplementary Figure S3A**). Next, an LDH-release assay was used to evaluate cytotoxicity. On average, HeLa cell viability remained above 90% after 24 h of treatment with each compound at 10 µM (**Supplementary Figure S3B**). The host-directed effects of the hit compounds were validated in MRSA-infected HeLa cells through classical CFU assays. Among the 17 hit compounds tested, 11 reduced the intracellular bacterial burden by more than 25%, including five 4-anilino-quinazolines (**Figure 2A**). Three compounds reduced the intracellular bacterial burden by over 50% and with statistical significance, namely GSK2008607A (56%; *p* = 0.007), GW296115X (67%; *p* = 0.001), and GW633459A (59%; *p* =0.02), and these were selected for further analyses.

Previously, it has been described that some small molecule drugs, in particular CADs, may accumulate in intracellular acidic compartment and exert their antimicrobial effect at locally increased concentrations (Schump et al., 2017; Tummino et al., 2021). This accumulation of CADs generally coincides with drug-induced lysosomal dysfunction and accumulation of phospholipids (Muehlbacher et al., 2012; Tummino et al., 2021). In contrast to amiodarone, a known CAD, the identified compounds did not classify as CADs based on calculated pKa and logP values (**Supplementary Figure S4A**). Furthermore, we did not find any evidence of lysosomal dysfunction using based on a NBD-PE incorporation assay (**Supplementary Figure S4B**). To fully exclude any direct antibiotic effects, the three compounds were added to planktonic MRSA cultures up to 100 μM concentration, which did not negatively affect bacterial growth (**Supplementary Figure S5**).

Compounds GSK2008607A, GW296115X and GW633459A were tested in other human cell lines infected with MRSA, including MelJuSo melanoma cells, A549 alveolar adenocarcinoma cells, MCF7 mammary gland adenocarcinoma cells and PMA-differentiated THP-1 macrophage-like cells (**Figures 2B–F**). There was significant variation in the susceptibility of the cell lines to MRSA, with MCF7 cells being the most susceptible (1.1x10^5^ CFUs/well in the DMSO control) and MelJuSo cells being the least susceptible (1.5x10^3^ CFUs/well in the DMSO control). The most effective compound, GW296115X, resulted in a statistically significant reduction in bacterial burden in HeLa (70% reduction, *p* = 0.0043), MelJuSo (88% reduction, *p* = 0.0019), and MCF7 cells (75% reduction, *p* = 0.0003). Compounds GSK2008607A and GW633459A were not active in A549 cells, but led to reductions in CFU counts in HeLa, MelJuSo, MCF7 and THP-1 cells, which was statistically significant in HeLa cells (72% reduction, *p* = 0.0017 for GSK2008607A and 50% reduction, *p* = 0.0043 for GW633459A) and MelJuSo cells (81% reduction, *p* = 0.039 for GSK2008607A and 81% reduction, *p* = 0.016 for GW633459A). In all cell lines the viability of the cells was equal or higher after treatment compared to the DMSO control (**Figures 2G–K**). GW296115X rescued the loss of viability of MRSA-infected HeLa (*p* = 0.0004) and A549 cells (*p* = 0.0003). Although GSK2008607A significantly improved the overall survival of MCF7 cells (*p* = 0.016), cytopathogenic effects were observed by light microscopy, including detachment and rounding of HeLa and THP-1 cells, vacuole formation in A549 cells and cell-cell fusion of MelJuSo cells (**Supplementary Figure S6**). GW296115X led to slight changes in cell morphology as well, with HeLa cells exhibiting a stretched morphology and THP-1 cells developing long dendrites, but these were not necessarily considered cytopathogenic. Changes in cell morphology were not observed after treatment with GW633459A. Taken together, compounds GSK2008607A, GW633459A and GW296115X were active in a broad range of infected cell lines, and in some cases rescued the loss in cell viability caused by MRSA infection.

### Dabrafenib analog GSK2008607A is a highly promiscuous kinase inhibitor that results in cytopathogenic effects

**Figure 3:**
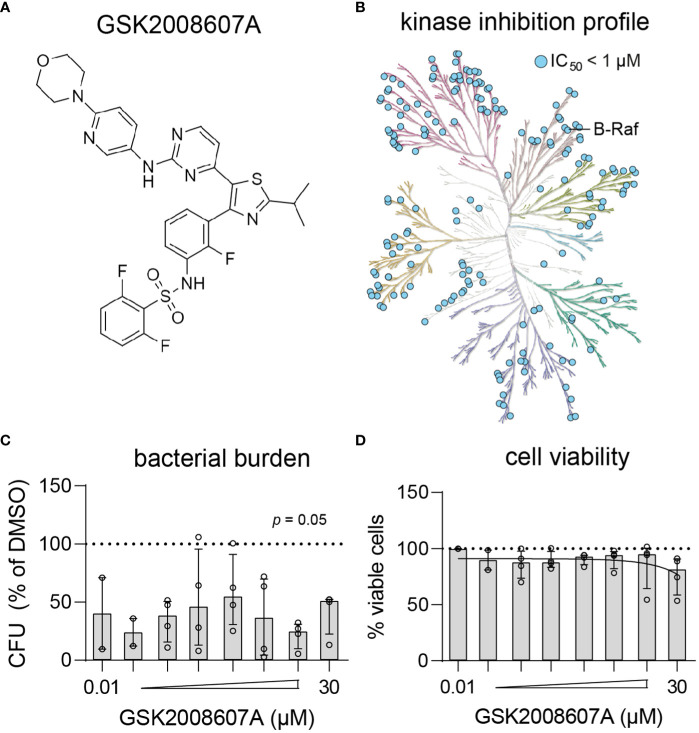
Kinase inhibition profile and potency of GSK2008607A. **(A)** Chemical structure of GSK2008607A. **(B)** The KinMap kinase phylogenetic tree shows the kinase inhibition profile of GSK2008607A as determined by KinomeScan (Drewry et al., 2017). Each circle represents a kinase target that is inhibited > 50% at 1 µM concentration. **(C, D)** GSK2008607A was tested at different concentrations to determine its antimicrobial potency **(C)** and level of cytotoxicity **(D)** in MRSA-infected HeLa cells. Bars and error bars show the median ± interquartile range. Each datapoint represents the results of an independent experiment. Statistical significance of observed differences was tested in comparison to the DMSO controls using Friedman tests.

Compound GSK2008607A (**Figure 3A**) is a dabrafenib analog synthesized during the development of a B-Raf^V600E^ inhibitor to treat advanced melanoma and non-small-cell lung cancers with this mutation (Stellwagen et al., 2011). In the present study, the compounds were initially screened at a concentration of 10 µM, at which GSK2008607A acts as a highly promiscuous kinase inhibitor (**Figure 3B**), which may explain its cytopathogenic effects. However, titration of GSK2008607A showed that it acts against intracellular MRSA at a concentration as low as 10 nM, at which the compound may target a more limited number of kinases (**Figures 3C**). At a concentration of 30 µM, GSK2008607A became cytotoxic as determined by LDH-release, but cytopathogenic effects were already observed in HeLa cells at 1 µM (**Figure 3D**).

### Lapatinib analog GW633459A is a pan-HER kinase inhibitor, but inhibits intracellular MRSA independently of the HER kinase family

**Figure 4:**
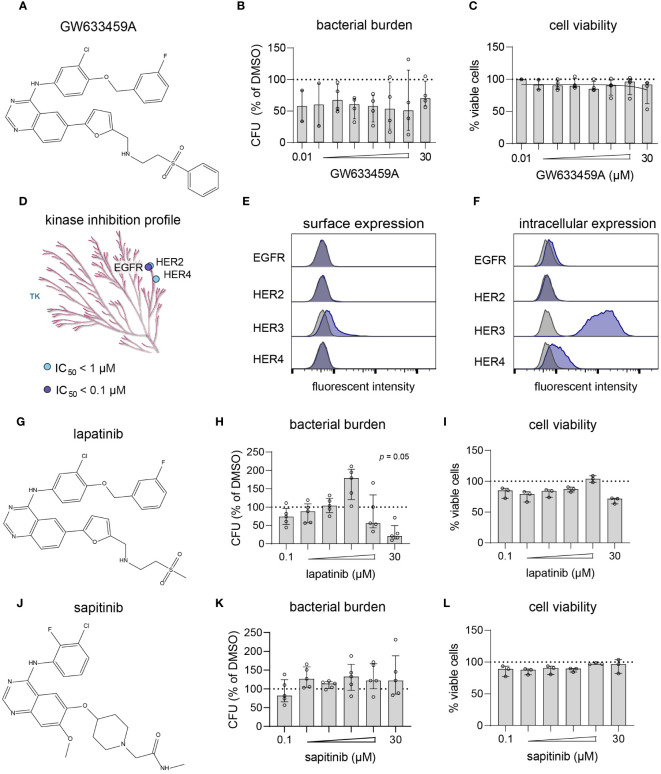
GW633459A is an inhibitor of the HER kinase family and reduces intracellular MRSA burden, but probably through a mechanism unrelated to these host kinase targets. **(A)** Chemical structure of GW633459A. **(B, C)** GW633459A was tested at different concentrations to determine its antimicrobial potency **(B)** and level of cytotoxicity **(C)** in MRSA-infected HeLa cells. **(D)** The KinMap kinase phylogenetic tree shows the kinase inhibition profile of GW633459A as determined by Caliper assay (Elkins et al., 2016). Each circle represents a kinase target that is inhibited > 50% at 0.1 µM (purple) or 1 µM (blue) concentration. Only the tyrosine kinase (TK) branch of the phylogenetic tree contains targets of GW633459A. **(E, F)** Baseline expression of the HER kinase family in HeLa cells was determined by flow cytometry after surface staining **(E)** or intracellular staining **(F)**. Kinase expression is shown in purple and isotype controls are shows in grey. **(G)** Chemical structure of lapatinib. **(H, I)** Lapatinib was tested at different concentrations to determine its antimicrobial potency **(H)** and level of cytotoxicity **(I)** in MRSA-infected HeLa cells. **(J)** Chemical structure of sapitinib. **(K, L)** Sapitinib was tested at different concentrations to determine its antimicrobial potency **(K)** and level of cytotoxicity **(L)** in MRSA-infected HeLa cells. Bars and error bars show the median ± interquartile range. Each datapoint represents the results of an independent experiment. Statistical significance of observed differences was tested in comparison to the DMSO controls using Friedman tests.

Next, compound GW633459A (**Figure 4A**) was titrated to determine its cytotoxicity and potency profile. The compound showed a large therapeutic window with efficacy observed at concentrations as low as 10 nM, while cell viability was only marginally affected at 30 µM (**Figures 4B, C**). GW633459A is a lapatinib analog synthesized during the development of a EGFR/HER2 dual inhibitor to treat breast cancer (Petrov et al., 2006). In addition, lapatinib and GW633459A are described to target HER4 at 1 µM concentration (**Figure 4D**) (Wood et al., 2004; Elkins et al., 2016). Since HER3 is catabolically inactive (Citri and Yarden, 2006), both lapatinib and GW633459A are pan-HER kinase inhibitors at 1 µM concentration. However, the involvement of the HER kinase family in the HDT effect of GW633459A seems unlikely based on several observations. Firstly, GW633459A was active in MRSA-infected HeLa cells that only expressed catabolically inactive HER3 on their surface (**Figure 4E**). Intracellularly, HeLa cells expressed both HER3 and HER4, however GW633459A was not active in MRSA-infected A549 cells that showed a similar HER3/4 expression pattern (**Figures 2**, **4F** and **Supplementary Figure S7**). Secondly, lapatinib, a structurally-related dual EGFR/HER2 inhibitor was only modestly active against intracellular MRSA at 10 and 30 µM concentrations (**Figures 4G–I**), while lapatinib was found detrimental for the host at 3 µM concentration, resulting in an 80% increase in the intracellular bacterial burden (**Figure 4H**). Lapatinib has been reported to result in direct antimicrobial effects at concentrations above 10 µM, especially against MRSA biofilm formation (Liu et al., 2022), although we did not observe this direct effect in planktonic MRSA cultures up to 100 µM concentration (**Supplementary Figure S5**). These results further indicate that EGFR/HER2 are not required for the HDT activity of GW633459A. Thirdly, sapitinib, another highly potent pan-HER kinase family inhibitor (Hickinson et al., 2010), was unable to reduce the bacterial burden of MRSA-infected HeLa cells (**Figures 4J–L**). Finally, a comparison of GW633459A with other 4-anilinoquinazolines showed that several 7-position substitutions (i.e., O-atom in the furanyl linker, thiazolyl linker, or terminal thiomorpholine group) resulted in reduced activity or no activity against intracellular MRSA, while their capacity to inhibit the HER kinase family remains largely unaffected (**Supplementary Table S4**) (Petrov et al., 2006; Elkins et al., 2016; Drewry et al., 2017). The target of GW633459A thus remains unclear, but is unlikely to be a member of the HER kinase family.

### GW296115X is a staurosporine-derivative with an improved kinase selectivity profile, and acts on intracellular MRSA by stimulating AMPK activation and enhancing autophagy

**Figure 5:**
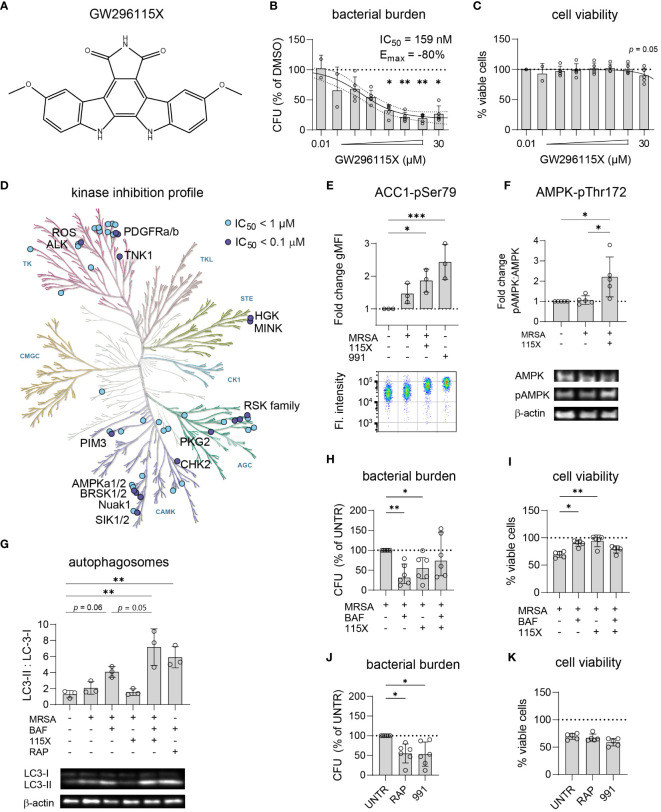
GW296115X activates AMPK resulting in autophagosome formation and elimination of intracellular MRSA. **(A)** Chemical structure of GW296115X. **(B, C)** GW296115X was tested at different concentrations to determine its antimicrobial potency **(B)** and level of cytoxicity **(C)** in MRSA-infected HeLa cells. **(D)** The KinMap kinase phylogenetic tree shows the kinase inhibition profile of GW296115X as determined by Caliper assay (Elkins et al., 2016). Each circle represents a kinase target that is inhibited > 50% at 0.1 µM (purple) or 1 µM (blue) concentration. **(E)** Phosphorylation of ACC1 at Ser80, a direct target of AMPK, was determined by flow cytometry and expressed as fold change in geometric mean fluorescent intensity (gMFI) compared to untreated HeLa cells. The AMPK-activating compound 991 was used as a positive control. **(F)** Phosphorylation of AMPK at Thr172 was determined by western blot and expressed as the ratio between phosphorylated and unphosphorylated AMPK. **(G)** Autophagosome formation was determined by western blot and expressed as the ratio between lipidated, membrane-bound LC3 (LC3-II) and cytosolic LC3 (LC3-I). Abbreviations: BAF = bafilomycin A1; RAP = rapamycin. The combination of lysosome inhibitor bafilomycin A1 and mTOR -inhibitor rapamycin results was used as a positive control that results in LC3-II accumulation. **(H, I)** The interaction between bafilomycin A1 and GW296115X and their effect against intracellular MRSA **(H)** and host cell viability **(I)** were determined. **(J, K)** The effect of two other autophagy-inducing compounds 991 and rapamycin against intracellular MRSA **(J)** and host cell viability **(K)** were determined. Each datapoint represents the results of an independent experiment. Statistical significance of observed differences was tested in comparison to the DMSO controls (*p < 0.05; **p < 0.01; ***p < 0.001).

Titration of compound GW296115X revealed its ability to reduce the intracellular bacterial burden of MRSA-infected HeLa cells by up to 80% with an IC_50_ of 159 nM (**Figures 5A, B**). In addition to its effect on the MRSA LAC JE2 strain, used for the PKIS screen, compound GW296115X was also found active against other strains of *S. aureus* (**Supplementary Figure S8A**). At 30 µM a limited cytotoxicity was observed which coincided with clear crystal formation (**Figure 5C**). Derived from the potent non-selective kinase inhibitor staurosporine, GW296115X exhibits much better selectivity, primarily targeting the type III receptor tyrosine kinase (RTK) family, the ribosomal S6 kinase (RSK) family, 5’ adenosine monophosphate-activated protein kinases (AMPK) and AMPK-related kinases BRSK1, BRSK2, and NuaK1 (**Figure 5D**) (Spacey et al., 1998; Elkins et al., 2016).

Using a bioluminescent strain of MRSA, we observed that GW296115X acts rapidly and the compound seems to both stimulate bacterial elimination and to impair its ability to replicate (**Supplementary Figure S8B**). As AMPK was previously described to become activated in MRSA-infected HeLa cells (Bravo-Santano et al., 2018), efforts to unravel the mechanism of action of GW296115X focused on AMPK and concomitant signaling. First, we determined the level of phosphorylation of ACC1 at Ser80, a downstream phosphorylation site of active AMPK and thus a readout for AMPK activity (Ross et al., 2017). Our initial hypothesis was that the level of AAC1 phosphorylation would increase upon infection with MRSA, followed by a decrease upon addition of the kinase inhibitor GW296115X. Indeed, ACC1 phosphorylation increased in the presence of MRSA, but this increase was statistically non-significant and limited as compared to cells stimulated with AMPK agonist 991 as a positive control (**Figure 5E**). Unexpectedly, addition of GW296115X resulted in increased ACC1 phosphorylation in MRSA-infected HeLa cells to a level that was significantly higher than untreated HeLa cells. Next, we determined the level of phosphorylation of AMPK at Thr172, an activating phosphorylation site. In agreement with the results of ACC1 phosphorylation, GW296115X significantly increased AMPK phosphorylation at Thr172 of MRSA-infected HeLa cells (**Figure 5F**). Combined, these results show that while GW296115X was previously found to act as an AMPK inhibitor in a biochemical assay (Elkins et al., 2016), this compound acts as an AMPK activator in our cellular assay.

AMPK plays a central role in maintaining cellular energy homeostasis. One AMPK-dependent mechanism to generate energy is the induction of autophagy by activation of ULK1, a central component in autophagy (Klionsky et al., 2021). Autophagy is also important for clearance of invasive bacteria from the cytosol, which is referred to as xenophagy (Klionsky et al., 2021). We measured autophagosome formation by LC3 conversion from its cytosolic form LC3-I into its lipidated form LC3-II, which is incorporated into autophagosomes. Upon MRSA infection, formation of LC3-II slightly increased (**Figure 5G**), which was not affected following treatment with GW296115X. In contrast, treating MRSA-infected cells with GW296115X in the presence of bafilomycin resulted in a strong accumulation of LC3-II, which extended beyond the level reached when MRSA-infected cells were treated only with bafilomycin A1 (*p* = 0.05). This demonstrated that GW296115X increased autophagic flux, most likely through an AMPK-dependent mechanism.

To determine whether the enhanced autophagic flux after GW296115X treatment was pivotal for its HDT effects, the flux was again blocked using bafilomycin A1 to assess effect on bacterial elimination. Indeed, adding bafilomycin A1 to treatment with GW296115X reverted both bacterial killing and increased host cell viability (**Figures 5H, I**). Surprisingly treatment with bafilomycin A1 alone led to 70% reduction in the intracellular bacterial burden. Thus, Bafilomycin A1 and GW296115X were both active against intracellular MRSA, but seem to antagonize each other. Finally, both compound 991, an allosteric AMPK activator (Lai et al., 2014), and rapamycin, an inhibitor of the mTOR complex (Klionsky et al., 2021), were able to reduce the intracellular MRSA burden similar to GW296115X (**Figures 5E, G, J**). However, only GW296115X was able to fully restore the loss in cell viability of MRSA-infected cells (**Figure 5K**). Of note, 991 also shows direct antibiotic effects against MRSA, which was not the case for bafilomycin A1, rapamycin or GW296115X (**Supplementary Figure S5**). Taken together, these results support autophagic flux induction through AMPK activation as a mechanism of action by which compound GW296115X exerts its effect against intracellular MRSA.

### GW296115X is safe *in vivo* and improves the survival rate of MRSA-infected zebrafish embryos

**Figure 6:**
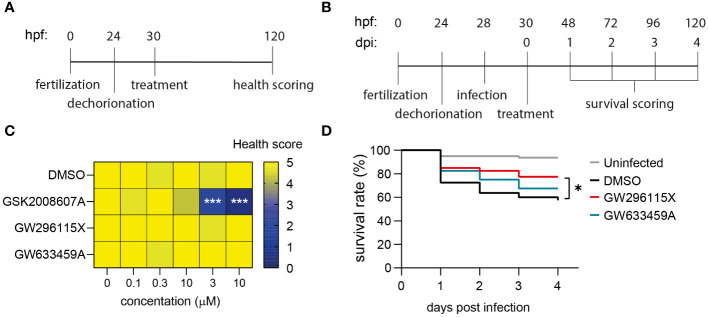
Safety and efficacy of PKIS hit compounds in *in vivo* zebrafish embryo models. **(A)** Zebrafish embryos were dechorionated 24 hours post fertilization (hpf), treated with compounds 30 hpf and scored for their health 120 hpf. Health score 5 = healthy embryos; for each of the following symptoms 1 point was subtracted: cranial malformations, oedema, irresponsiveness, tail curve malformations; health score 0 = death embryos. **(B)** Dechorionated zebrafish embryos were infected at 28hpf with 1000 CFUs MRSA and treated at 30 hpf. The survival of the embryos was assessed daily by checking their heart beat. **(C)** Dechorionated zebrafish embryos were treated with serial dilutions of DMSO, GSK2008607A, GW296115X, and GW633459A to assess health parameters (n = 15 for each treatment and concentration). **(D)** Infected zebrafish embryos were treated with 10 µM GW296115X (n = 40), 10 µM GW633459A (n = 40) or 0.1% DMSO as negative solvent control (n = 80). Uninfected controls were included to assess the health state of the embryos at baseline. The data were collected in two independent experiments. Differences between treated and untreated (i.e., DMSO) groups were tested for statistical significance (*p < 0.05, ***p < 0.001).

In order to investigate the *in vivo* effects of GSK2008607A, GW633459A and GW296115X, we utilized zebrafish embryo toxicity and efficacy models. The compounds were added to dechorionated zebrafish embryos and incubated for four days to assess toxicity (**Figure 6A**; **Supplementary Figure S9**). After 4 days, 2/75 zebrafish embryos died in the DMSO control group and no further signs of toxicity were observed among the surviving embryos (**Figure 6C**). In contrast, compound GSK2008607A caused zebrafish embryo mortality at 3 and 10 µM. Surviving zebrafish embryos exhibited oedema of the heart sac and yolk, as well as unresponsiveness to physical stimulation. Of the 15 zebrafish embryos exposed to GSK2008607A at 1 µM, one died, another did not respond to physical stimulation, two zebrafish embryos showed cranial malformations and three zebrafish embryos displayed oedema. In contrast, the mortality rate for compounds GW296115X and GW633459A was similar to the DMSO control, and no further signs of toxicity were observed.

The efficacy of the compounds was determined in a zebrafish embryo MRSA infection model. At 28 hours post fertilization dechorionated embryos were injected in the blood island with 1000 CFUs MRSA and their survival was monitored for four days (**Figure 6B**). We used the 1 µM GSK2008607A for treating MRSA-infected zebrafish embryos, which was expected to be a safe concentration based on the toxicity model. However, the combination of the compound and MRSA infection caused severe compound-related toxicity early on in the efficacy experiment. MRSA infection led to a survival rate of 57.5% after 4 days, which was significantly lower than 93.8% in uninfected controls (**Figure 6D**). Compound GW633459A moderately increased the survival rate to 67.5%, but this effect was not statistically significant (*p* = 0.27). Finally, compound GW296115X increased the survival rate to 77.5% with statistical significance (*p* = 0.03), showing that compound GW296115X is safe and active against MRSA both *in vitro* and *in vivo* zebrafish model.

## Discussion

The increasing incidence of MRSA infections and their growing resistance to last-resort antibiotics demands novel therapeutic approaches (Lehar et al., 2015; Cassini et al., 2019; Shariati et al., 2020; Murray et al., 2022). HDTs targeting host kinases have recently emerged as a promising strategy for treatment of MRSA infections (Bravo-Santano et al., 2019a; Bravo-Santano et al., 2019b). In this study, we conducted a screen of 201 kinase inhibitors selected from the PKIS library, based on previous efficacy in intracellular infection models with *Salmonella enterica* Typhimurium and *Mycobacterium tuberculosis* (van den Biggelaar et al., 2023), to identify potential HDTs for the treatment of intracellular MRSA infection. The screen resulted in 17 hit compounds that reduced the intracellular MRSA burden of infected HeLa cells of which the most effective compounds were selected for further examination: GSK2008607A, GW296115X, and GW633459A. These compounds demonstrated broad efficacy in MRSA-infected cell lines derived from diverse tissue origins. While GW296115X and GW633459A exhibited negligible cytotoxicity in these cell lines, treatment with GSK2008607A led to cytopathogenic effects. In zebrafish embryos, compounds GW296115X and GW633459A were well tolerated up to a concentration of 10 µM, in contrast to GSK2008607A, which exhibited toxicity at concentrations ≥ 1 µM. Importantly, both GW296115X and GW633459A improved survival of MRSA-infected zebrafish embryos, with GW296115X displaying statistically significant results.

Compound GW633459A, as well as many other 4-anilinoquinozalines from the PKIS library, shares structural similarity with lapatinib, an FDA-approved drug to treat HER2-positive breast cancer. Given that some MRSA virulence factors are known to indirectly activate EGFR signaling (Lemjabbar and Basbaum, 2002; Gómez et al., 2007; Breshears et al., 2012; Breshears et al., 2016), and considering that both GW633459A (Elkins et al., 2016) and lapatinib (Petrov et al., 2006; Qiu et al., 2008) are pan-HER kinase family inhibitors, we hypothesized that the mechanism of action of GW633459A would likely involve the HER kinase family. However, MRSA-infected HeLa cells only expressed catabolically inactive HER3, which contradicts this hypothesis (Citri and Yarden, 2006). Furthermore, treatment with lapatinib or the more potent pan-HER kinase inhibitor sapitinib (Hickinson et al., 2010) had minimal effect on intracellular bacteria in our model. Analysis of the 4-anilinoquinazoline structure-activity relationship suggested that the host-beneficial effects of these compounds were dependent on their 7-position chemical groups. Interestingly, these moieties affect their bioavailability, but not their affinity or specificity for HER kinases (Lackey, 2006; Petrov et al., 2006; Elkins et al., 2016; Tamir et al., 2020). Consequently, the involvement of the HER family in impairing intracellular survival of MRSA by GW633459A seems unlikely. Lapatinib was recently described to directly act on MRSA and to affect biofilm formation (Liu et al., 2022), but we did also not observe any direct antibacterial effects for GW633459A or lapatinib up to 100 μM concentration, indicating that these kinase inhibitors act exclusively through host-directed effects. In conclusion, although GW633459A was described to specifically inhibit the HER kinase family, our study indicates that its antimicrobial effects rely on different host targets.

**Figure 7:**
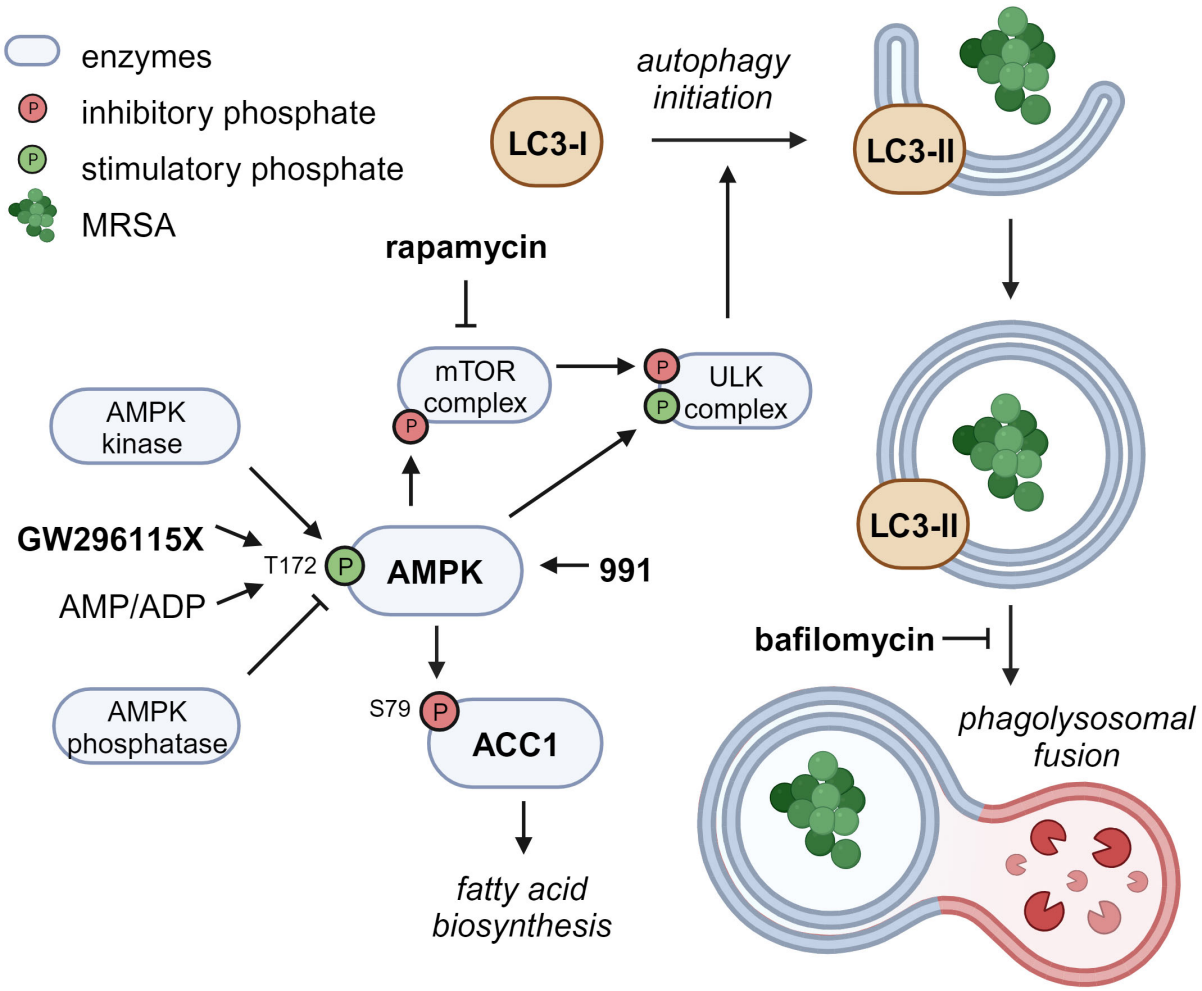
Proposed mechanism of action of compound GW296115X. Compound GW296115X either stimulates AMPK T172 phosphorylation or inhibits its dephosphorylation, resulting in activation of AMPK. Active AMPK phosphorylates ACC1, resulting in decreased fatty acid biosynthesis. In addition, active AMPK phosphorylates the mTOR complex and the ULK complex both directly, thereby inactivating the mTOR complex and activating the ULK complex, which facilitates the initiation of autophagy. The AMPK-activating 991 and mTOR-inhibitor rapamycin show similar effects on ACC1 phosphorylation and autophagy initiation, respectively. Bafilomycin blocks autophagosome degradation, thereby resulting in increased LC3-II levels, especially in combination with drugs that stimulate autophagy initiation including GW296115X or rapamycin. Created with BioRender.com.

Compound GW296115X, also known as 3744W, originates from staurosporine and initially gained attention because of its ability to potently block PDGF receptor autophosphorylation while displaying promising target selectivity (Spacey et al., 1998). Later, GW296115X was found to target several other kinase families, including the type III RTK family, the RSK family, AMPK and AMPK-related kinases (Elkins et al., 2016; Tamir et al., 2020). AMPK was previously found to be involved in host-pathogen interactions of MRSA-infected cells and could therefore be the prime target responsible for the antimicrobial effect of GW296115X (Kumar et al., 2016; Bravo-Santano et al., 2018). Activity of AMPK, a heterotrimeric complex, primarily relies on Thr172 phosphorylation within the activation loop of the α subunit, and is modulated by AMPK kinases (mainly LKB1 and CaMKK2), AMPK phosphatases, and allosteric binding of AMP and ADP to the γ-subunit (Oakhill et al., 2011). While GW296115X was previously found to inhibit AMPK in a biochemical assay (Elkins et al., 2016), we observed enhanced Thr172 phosphorylation and AMPK activity in cellular assays. Other predicted AMPK inhibitors that actually act as AMPK activators in practice have previously been described, including staurosporine (Chandrashekarappa et al., 2013; Li et al., 2016; Ross et al., 2017). These compounds bind to the ATP-binding pocket of the α subunit causing conformational changes that either promote Thr172 phosphorylation by upstream AMPK kinases, as described for SU6656 (Ross et al., 2017), or prevent Thr172 dephosphorylation by AMPK phosphatases, as described for staurosporine (Chandrashekarappa et al., 2013), both leading to prolonged AMPK activity that can only be observed in cellular assays. Based on structural similarity with staurosporine, compound GW296115X most likely prevents AMPK Thr172 dephosphorylation, thereby enhancing AAC1 phosphorylation and autophagosome formation via phosphorylation of mTOR and/or ULK1, resulting in their inhibition and activation, respectively (**Figure 7**) (Klionsky et al., 2021).

The role of autophagy in the elimination of intracellular *S. aureus* has been under debate, with some studies showing that autophagy benefits the host (Neumann et al., 2016; Gibson et al., 2021; Prajsnar et al., 2021), whereas others indicate that autophagy benefits the bacteria (Schnaith et al., 2007; Bravo-Santano et al., 2018). The latter studies may be confounded by measurements of LC3-associated phagocytosis (LAP) or endocytosis (LANDO) rather than selective autophagy (Peña-Martinez et al., 2023). Unlike selective autophagy, phagocytosis provides an intracellular niche for *S. aureus* replication (Flannagan et al., 2016; Prajsnar et al., 2021). Compound GW296115X most likely acts through selective autophagy by activating AMPK, whereas LAP and LANDO occur independently of AMPK or mTOR signaling (Losier et al., 2019). Combined treatment with autophagy inducer GW296115X and lysosome inhibitor bafilomycin A1 reverted the HDT effect of GW296115X, providing additional evidence that stimulation of the autophagy pathway by GW296115X is host beneficial. However, we observed a lower bacterial burden after treatment with bafilomycin A1 alone, as previously observed by others (Tranchemontagne et al., 2016; Lacoma et al., 2017). This may also be explained by its inhibitory effect on phagocytosis (Tranchemontagne et al., 2016; Jacquin et al., 2017; Hooper et al., 2022). LC3-associated pathways require further exploration to better understand their individual roles during intracellular *S. aureus* infection.

In summary, two potent kinase inhibitors, GW633459A and GW296115X were identified from the PKIS library to be used as HDT during MRSA infection. While the host target of GW633459A remains elusive and requires further investigation, we were able to identify AMPK as an important host kinase affected by GW296115X. GW296115X paradoxically enhanced AMPK activity, resulting in enhanced autophagosome formation and bacterial degradation. Interestingly, GW296115X was previously found to show efficacy against intracellular *Mycobacterium abcessus*, making it plausible that this compound could be broadly applicable to treat bacterial infections (Adrian et al., 2019). Collectively, our findings *in vitro* and *in vivo* demonstrate the potential of PKIS compounds GW296115X and GW633459A as promising for further development as HDTs against intracellular MRSA.

## Data availability statement

The original contributions presented in the study are included in the article/**Supplementary Material**. Further inquiries can be directed to the corresponding authors.

## Ethics statement

Ethical approval was not required for the studies on humans in accordance with the local legislation and institutional requirements because only commercially available established cell lines were used. The animal study was approved by Animal Welfare Body of Leiden University. The study was conducted in accordance with the local legislation and institutional requirements.

## Author contributions

RvdB: Conceptualization, Formal analysis, Investigation, Methodology, Visualization, Writing – original draft, Writing – review & editing. KW: Investigation, Methodology, Writing – review & editing. SvdE: Investigation, Methodology, Writing – review & editing. CvD: Investigation, Methodology, Writing – review & editing. EM: Resources, Supervision, Writing – review & editing. AdR: Investigation, Methodology, Writing – review & editing. AM: Funding acquisition, Supervision, Writing – review & editing. TO: Funding acquisition, Supervision, Writing – review & editing. AS: Conceptualization, Project administration, Supervision, Writing – review & editing.

## Supplementary material

Supplementary Table 2 Raw values and z-scores for MRSA burden (z-score_MRSA+_) and HeLa cell count (z-score_HeLa_) as measured in the flow cytometry screen.

Supplementary Table 3 Information about the PKIS hit compounds.

Supplementary Table 4 Structure-activity relationship of PKIS 4-anilinoquinazolines with inhibition of MRSA in infected HeLa cells and inhibition of the HER kinase family.

Supplementary Figure 1 Characteristics of the MRSA intracellular infection model. **(A)** Histogram demonstrating GFP expression by USA300 LAC JE2 strain, as compared to wildtype BK 11540 strain. **(B)** Representative example of percentage of infected HeLa cells exposed to live and heat-inactivated bacteria, at 1 and 24 h post-infection. **(C, D)** Histograms corresponding to the results shown in **(B)**. **(E)** Growth curves of bioluminescent MSRA after infection of HeLa cells, in the absence or presence of 5 µg/ml gentamicin. **(F)** Representative images of uninfected HeLa cells, infected HeLa cells treated with 5 µg/ml gentamicin and infected HeLa cells that did not receive any treatment. White arrow depicts lytic host cell with escaping bacteria.

Supplementary Figure 2 Chemical structures of the 18 PKIS hit compounds. Compounds part of 4-anilino-quinazoline or 4-pyrimidinyl ortho-aryl azole chemotypes are indicated by purple and red boxes, respectively.

Supplementary Figure 3 Validation of selected PKIS hit compounds. **(A)** Potential direct antimicrobial effects of the selected PKIS compounds were assessed in cell-free, planktonic MRSA cultures, and OD600 measurements were performed to determine effects on bacterial growth, expressed as a percentage of the DMSO control. **(B)** The *in vitro* safety of the compounds was tested in an LDH-release assay. The cell viability of treated HeLa cells is given as the percentage of DMSO-treated HeLa cells.

Supplementary Figure 4 Prediction and experimental validation of cationic amphiphilic drugs. **(A)** Prediction of compounds that act as cationic amphiphilic drugs based on their calculated pKa (≥ 7.4) and logP values

Supplementary Figure 5 Potential direct antimicrobial effects of GSK2008607A, GW633459A, GW296115X, lapatinib, 991, bafilomycin A1, and rapamycin were assessed in cell-free, planktonic MRSA cultures, and OD600 measurements were performed to determine effects on bacterial growth. The data are expressed as change in OD600 to correct for compound-related differences in turbidity at t = 0h. The experiment was performed in triplicate.

Supplementary Figure 6 Representative light microscopy images of HeLa, MelJuSo, A549, MCF7, and PMA-differentiated THP-1 cells treated with DMSO, GSK2008607A, GW296115X or GW633459.

Supplementary Figure 7 Expression of the HER kinase family in MCF7 and A549 cells. **(A, B)** Expression was determined for MCF7 cells by flow cytometry after surface staining **(A)** or intracellular staining **(B)**. **(C, D)** Expression was determined for A549 cells by flow cytometry after surface staining **(C)** or intracellular staining **(D)**. Kinase expression is shown in purple and isotype controls are shows in grey.

Supplementary Figure 8 Activity of GW296115X upon infection of HeLa cells with different strains of *S. aureus* and over time. **(A)** The efficacy of GW296115X against different strains of *S. aureus* was determined by CFU count and given as the percentage of the DMSO control. **(B)** Growth curves of bioluminescent MSRA after infection of HeLa cells, in the presence of 0.1% DMSO or 10 µM GW296115X.

Supplementary Figure 9 Representative images of healthy and malformed zebrafish embryos.
