# Supplementary material for: Identification of kinase modulators as host-directed therapeutics against intracellular methicillin-resistant Staphylococcus aureus
Source: Front Cell Infect Microbiol. 2024 Mar 25;14:1367938. doi: 10.3389/fcimb.2024.1367938 (PMC10999543; doi:10.3389/fcimb.2024.1367938)
Supplement: Supplementary file 1 [file Table_1.docx]

**Supplementary Table 1** List of antibodies used in this study.

| **Antibodies** | **Source** | **Concentration** |
| --- | --- | --- |
| Mouse anti-human EGFR (clone AY13) | BioLegend | 10 µg/ml |
| Mouse anti-human HER2 (clone 24D2) | BioLegend | 2.5 µg/ml |
| Mouse anti-human HER3 (clone RTJ2) | NOVUS Biologicals | 10 µg/ml |
| Mouse anti-human HER4 (Ab77) | ThermoFisher Scientific | 10 µg/ml |
| Mouse isotype control (clone MG1-45) | BioLegend | Equal concentration |
| Rabbit anti-human ACC1-pSer79 (clone D7D11) | Cell Signaling Technology | 4.2 µg/ml |
| Goat anti-mouse IgG-RPE | Agilent | 1:100 |
| Goat anti-rabbit IgG-AlexaFluor647 | Invitrogen | 1:400 |
| Mouse anti-human beta actin (clone AC-15) | Sigma-Aldrich | 1:10,000 |
| Rabbit anti-human LC3B | NOVUS Biologicals | 2 µg/ml |
| Rabbit anti-human AMPK | Cell Signaling Technologies | 88 ng/ml |
| Rabbit anti-human AMPK-pThr172 (clone 40H9) | Cell Signaling Technologies | 27 ng/ml |
| Goat anti-mouse-HRP | ThermoFisher Scientific | 200 ng/ml |
| Goat anti-rabbit-HRP | ThermoFisher Scientific | 200 ng/ml |
