# Supplementary material for: Identification of kinase modulators as host-directed therapeutics against intracellular methicillin-resistant Staphylococcus aureus
Source: Front Cell Infect Microbiol. 2024 Mar 25;14:1367938. doi: 10.3389/fcimb.2024.1367938 (PMC10999543; doi:10.3389/fcimb.2024.1367938)
Supplement: Supplementary Table 4 — Structure-activity relationship of PKIS 4-anilinoquinazolines with inhibition of MRSA in infected HeLa cells and inhibition of the HER kinase family. [file Table_4.docx]

**Supplementary Table 4** Properties of PKIS 4-anilino-quinazolines tested in the flow cytometry screen.

|  |  | |  | | | | | | | | | | | |
| --- | --- | --- | --- | --- | --- | --- | --- | --- | --- | --- | --- | --- | --- | --- |
| **compound** | |  | | **R1** | **R2** | **R3** | **R4** | **R5** | **z-score**  **GFP+** | **CFU (% of DMSO) at 10 µM** | **% inhibition at 1 µM** | | |  |
|  |  | **library** | |  |  |  |  |  |  |  | **EGFR** | **HER2** | **HER4** |  |
| GW574783B | | PKIS1 | | Cl | F |  | NH | 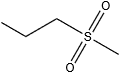 | -2.30 | 53.4 | 91 | 75 | 89 |  |
| GW580496A | | PKIS1 | | Cl | H |  | NH | 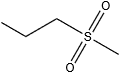 | -2.73 | 65.3 | 82 | 74 | 80 |  |
| GW616030X | | PKIS1 | | Br | H |  | NCH_2_CN | 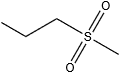 | -2.24 | 50.3 | 77 | 67 | 74 |  |
| GW621823A | | PKIS1 | | Cl | F |  | N(CH_2_)_2_CH_3_ | 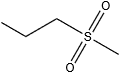 | -2.21 | 68.4 | 78 | 82 | 79 |  |
| GW633459A | | PKIS1 | | Cl | F |  | NH | 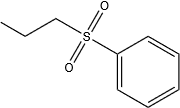 | -2.34 | 41.2 | 83 | 60 | 89 |  |
| GW576924A | | PKIS1 | | F | H |  | 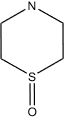 | | -2.36 | 137.9 | 82 | 91 | 75 |  |
| GW576609A | | PKIS1 | | Cl | F |  | 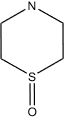 | | -1.46 |  | 84 | 80 | 91 |  |
| GW583373A | | PKIS1 | | Cl | H |  | 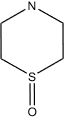 | | -1.31 |  | 77 | 73 | 84 |  |
| GW615311X | | PKIS1 | | Cl | F |  | O | 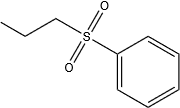 | -0.16 |  | 85 | 70 | 73 |  |
| GW582764 | | PKIS2 | | Cl | H |  | NH | 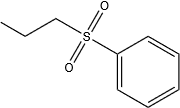 | -1.31 |  | 100 | 100 | 97.8 |  |
| GW583340 | | PKIS2 | | Cl | F | **** | NH | 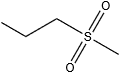 | -1.71 |  | 99.9 | 99.7 | 99.1 |  |
