# Supplementary figures and images for: Identification of kinase modulators as host-directed therapeutics against intracellular methicillin-resistant Staphylococcus aureus
Source: Front Cell Infect Microbiol. 2024 Mar 25;14:1367938. doi: 10.3389/fcimb.2024.1367938 (PMC10999543; doi:10.3389/fcimb.2024.1367938)

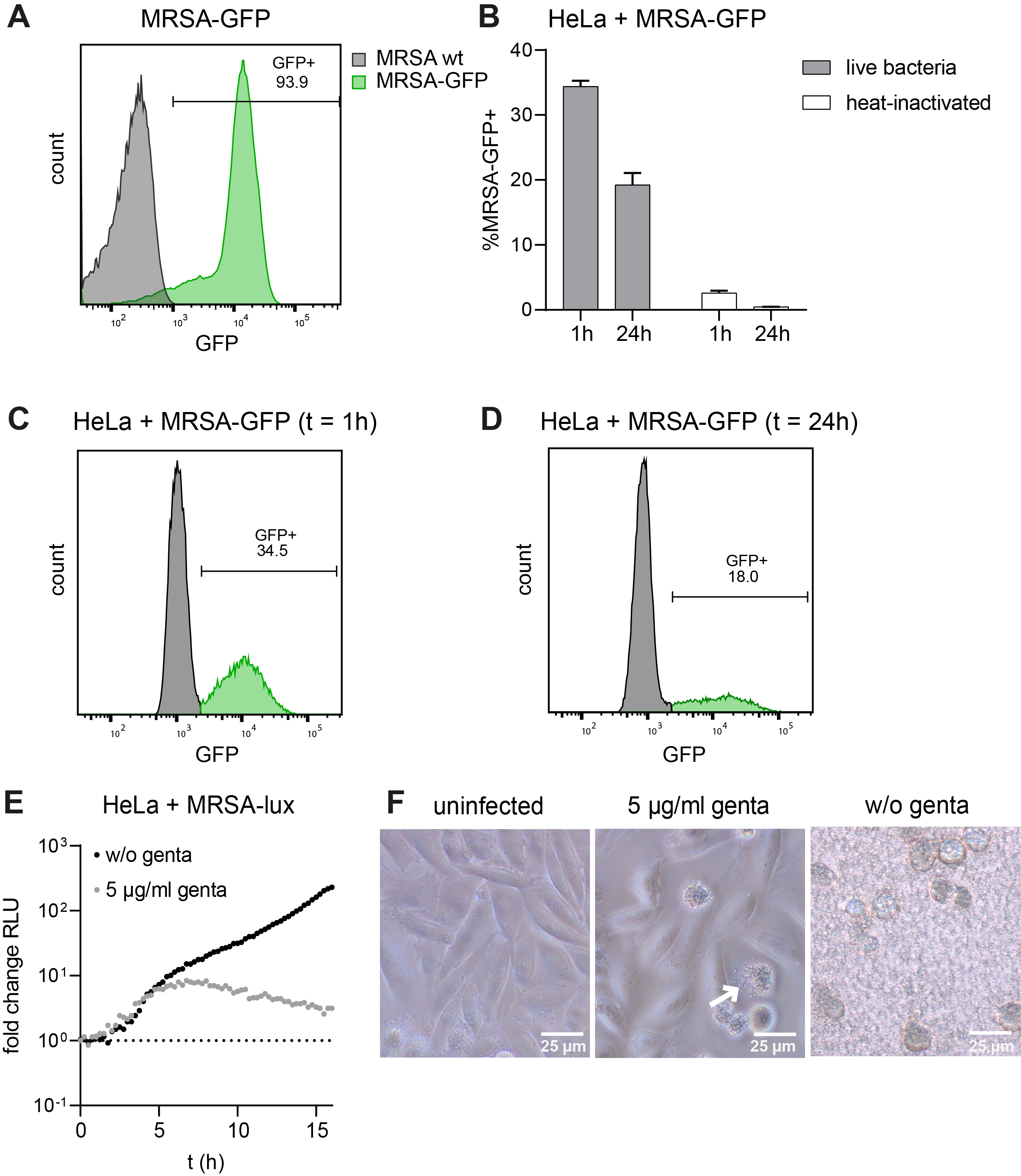

Supplement: Supplementary Figure 1 — Characteristics of the MRSA intracellular infection model. (A) Histogram demonstrating GFP expression by USA300 LAC JE2 strain, as compared to wildtype BK 11540 strain. (B) Representative example of percentage of infected HeLa cells exposed to live and heat-inactivated bacteria, at 1 and 24 h post-infection. (C, D) Histograms corresponding to the results shown in (B). (E) Growth curves of bioluminescent MSRA after infection of HeLa cells, in the absence or presence of 5 µg/ml gentamicin. (F) Representative images of uninfected HeLa cells, infected HeLa cells treated with 5 µg/ml gentamicin and infected HeLa cells that did not receive any treatment. White arrow depicts lytic host cell with escaping bacteria. [file Image_1.jpeg]

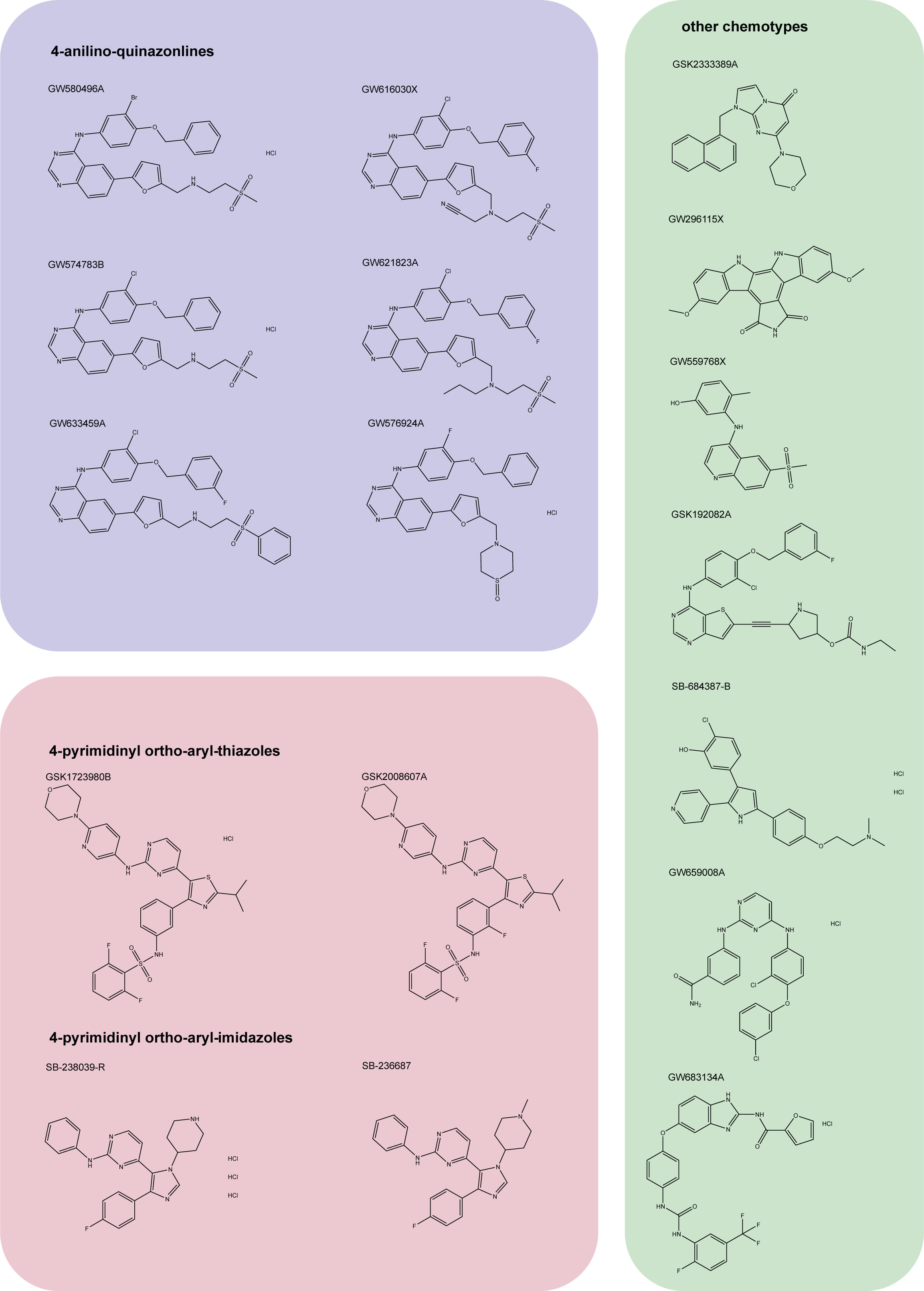

Supplement: Supplementary Figure 2 — Chemical structures of the 18 PKIS hit compounds. Compounds part of 4-anilino-quinazoline or 4-pyrimidinyl ortho-aryl azole chemotypes are indicated by purple and red boxes, respectively. [file Image_2.jpeg]

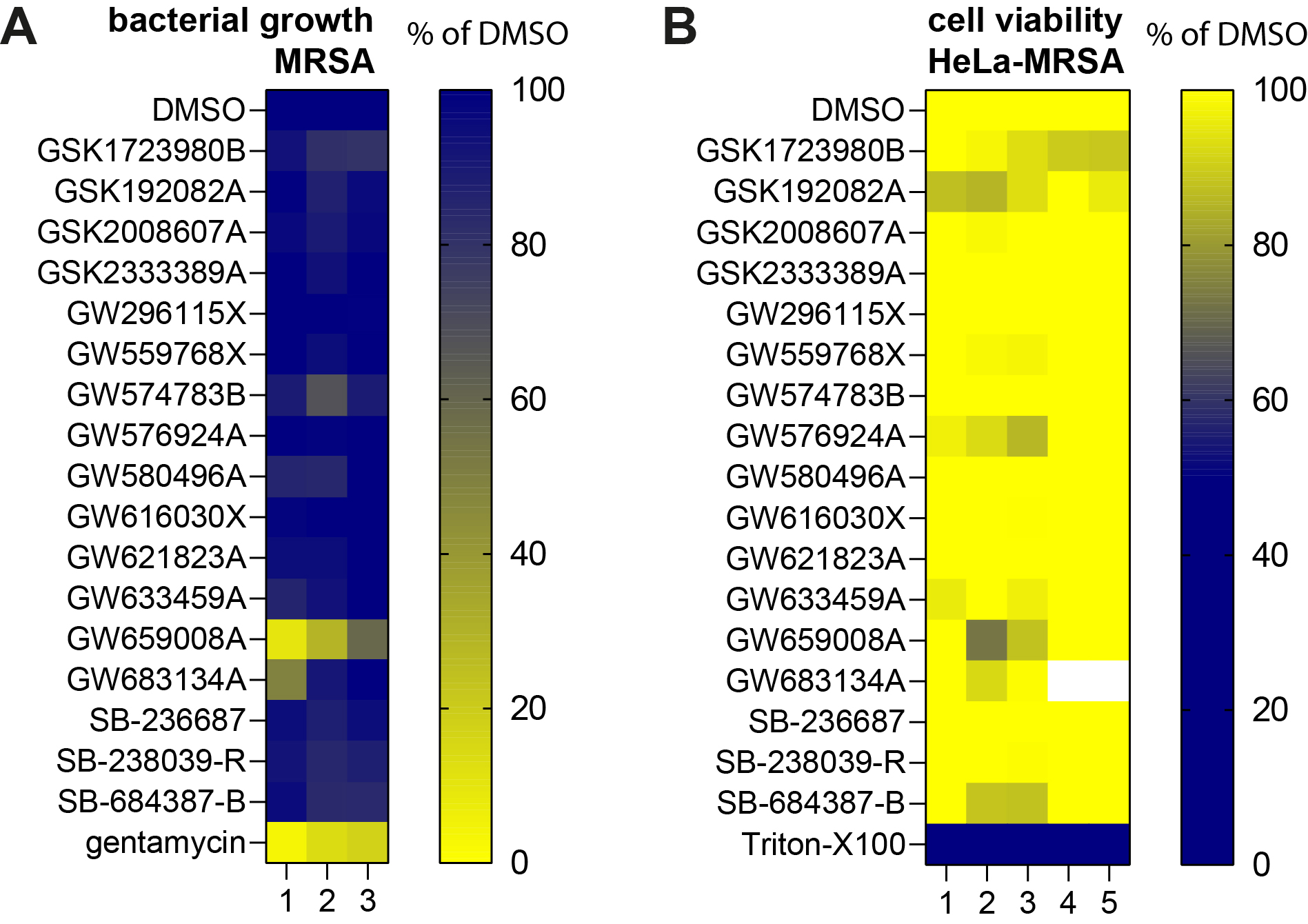

Supplement: Supplementary Figure 3 — Validation of selected PKIS hit compounds. (A) Potential direct antimicrobial effects of the selected PKIS compounds were assessed in cell-free, planktonic MRSA cultures, and OD600 measurements were performed to determine effects on bacterial growth, expressed as a percentage of the DMSO control. (B) The in vitro safety of the compounds was tested in an LDH-release assay. The cell viability of treated HeLa cells is given as the percentage of DMSO-treated HeLa cells. [file Image_3.jpeg]

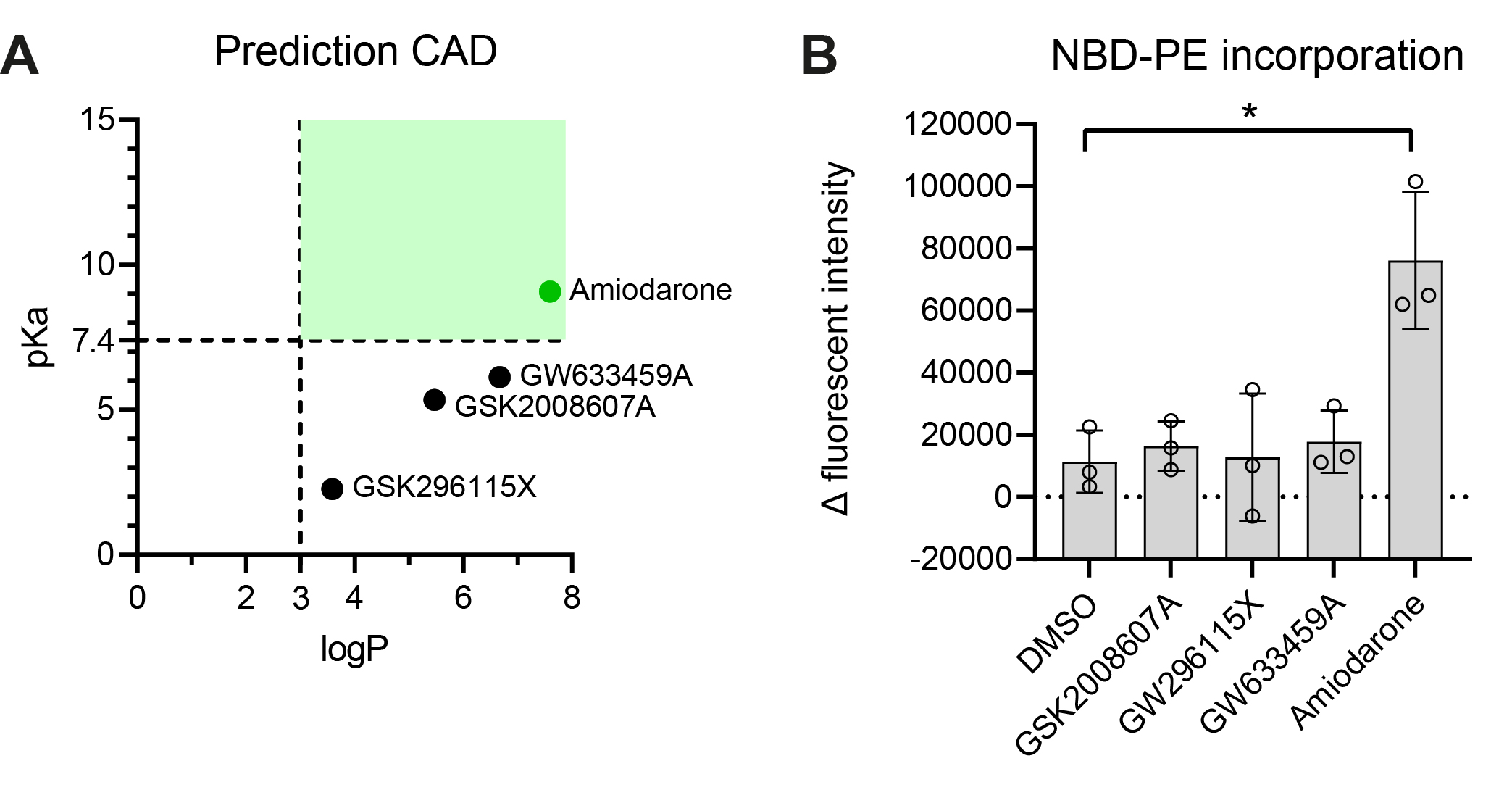

Supplement: Supplementary Figure 4 — Prediction and experimental validation of cationic amphiphilic drugs. (A) Prediction of compounds that act as cationic amphiphilic drugs based on their calculated pKa (≥ 7.4) and logP values [file Image_4.jpeg]

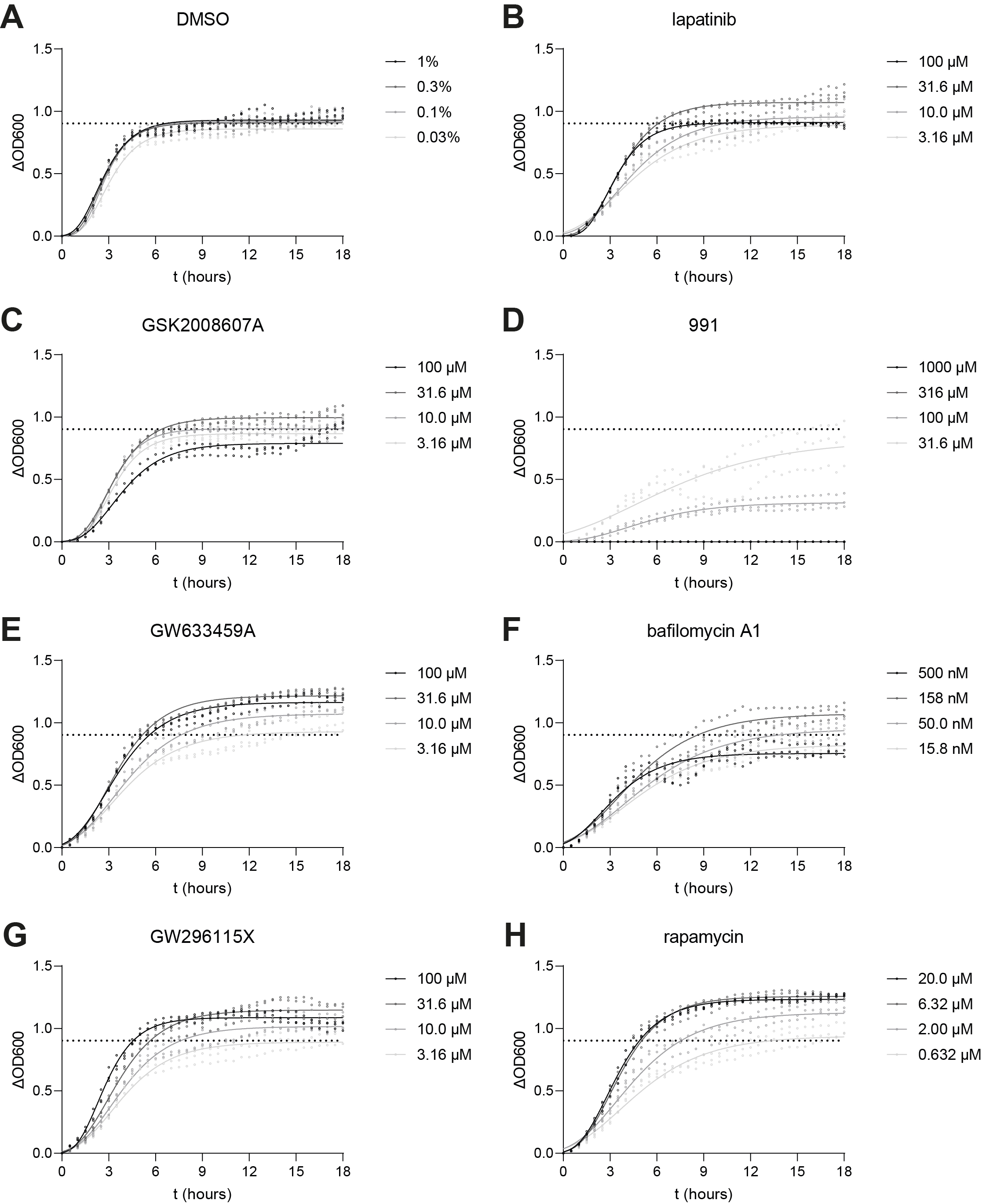

Supplement: Supplementary Figure 5 — Potential direct antimicrobial effects of GSK2008607A, GW633459A, GW296115X, lapatinib, 991, bafilomycin A1, and rapamycin were assessed in cell-free, planktonic MRSA cultures, and OD600 measurements were performed to determine effects on bacterial growth. The data are expressed as change in OD600 to correct for compound-related differences in turbidity at t = 0h. The experiment was performed in triplicate. [file Image_5.jpeg]

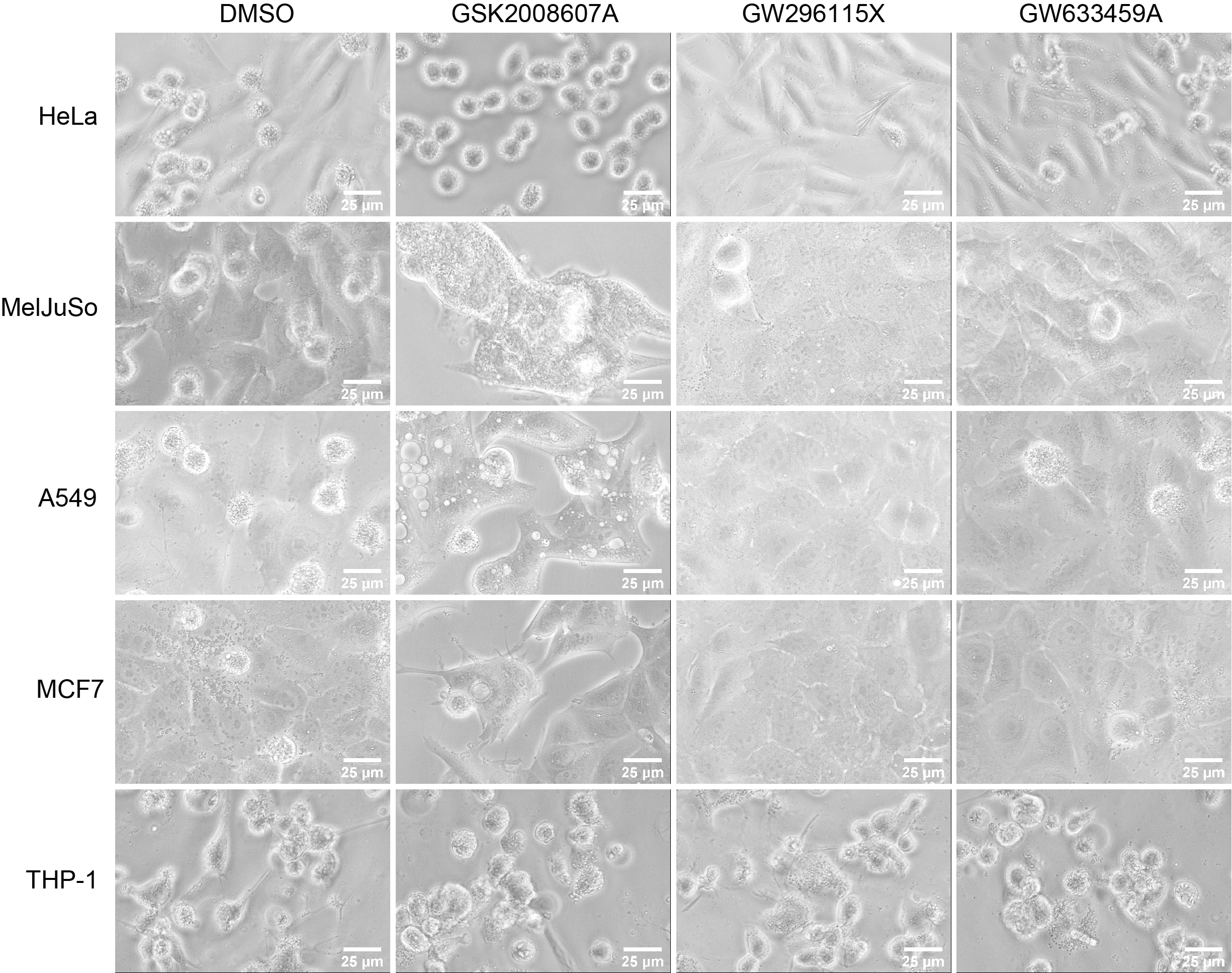

Supplement: Supplementary Figure 6 — Representative light microscopy images of HeLa, MelJuSo, A549, MCF7, and PMA-differentiated THP-1 cells treated with DMSO, GSK2008607A, GW296115X or GW633459. [file Image_6.jpeg]

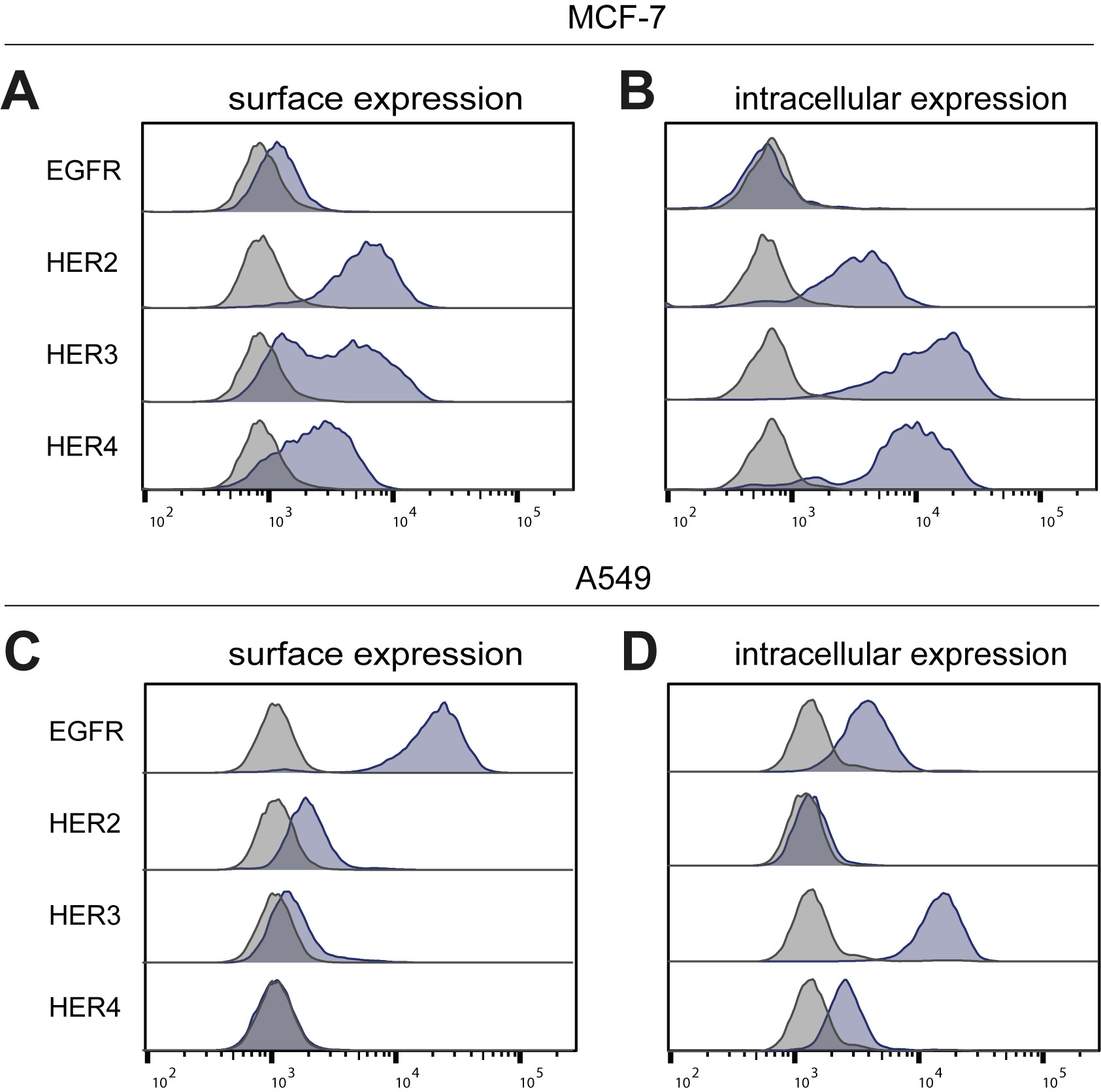

Supplement: Supplementary Figure 7 — Expression of the HER kinase family in MCF7 and A549 cells. (A, B) Expression was determined for MCF7 cells by flow cytometry after surface staining (A) or intracellular staining (B). (C, D) Expression was determined for A549 cells by flow cytometry after surface staining (C) or intracellular staining (D). Kinase expression is shown in purple and isotype controls are shows in grey. [file Image_7.jpeg]

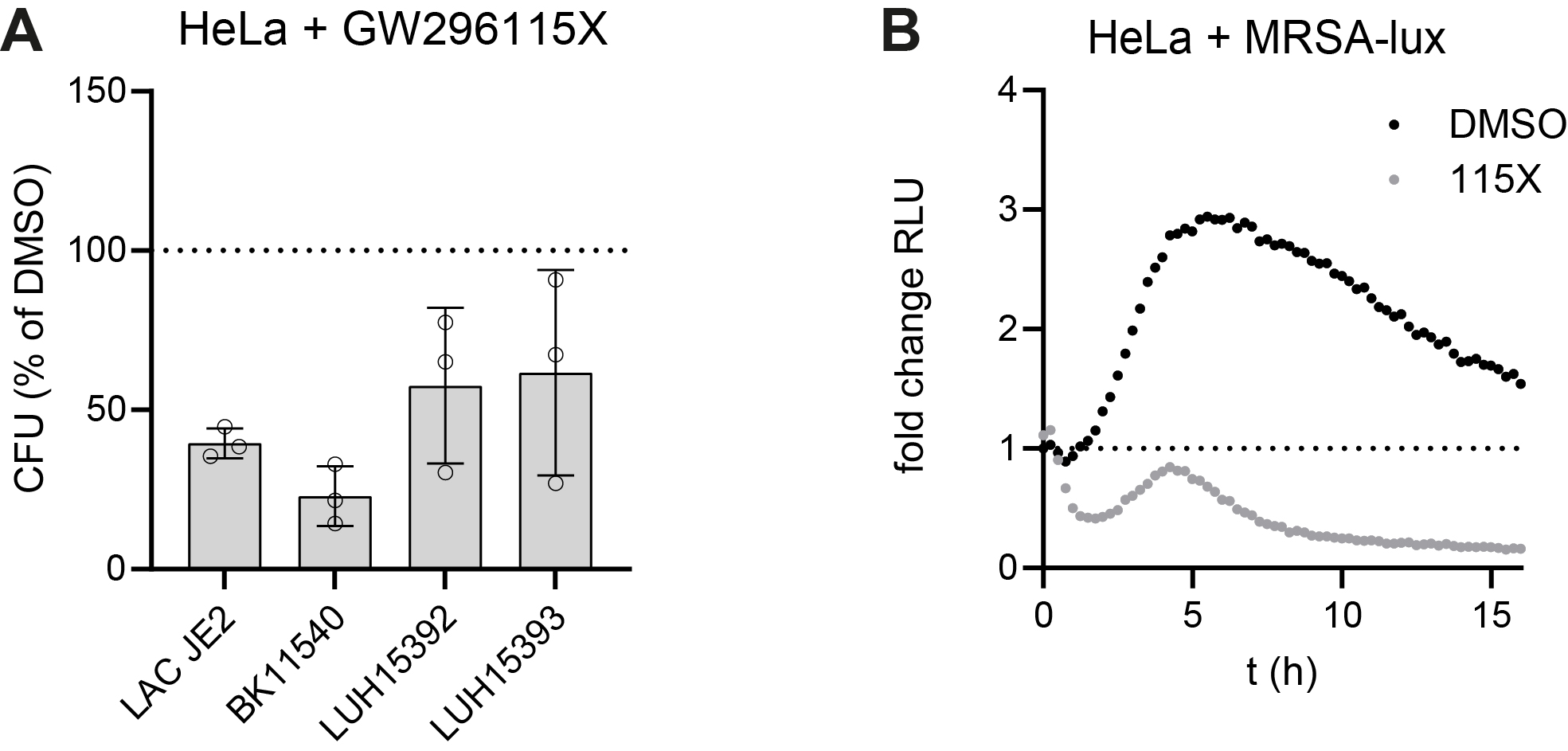

Supplement: Supplementary Figure 8 — Activity of GW296115X upon infection of HeLa cells with different strains of S. aureus and over time. (A) The efficacy of GW296115X against different strains of S. aureus was determined by CFU count and given as the percentage of the DMSO control. (B) Growth curves of bioluminescent MSRA after infection of HeLa cells, in the presence of 0.1% DMSO or 10 µM GW296115X. [file Image_8.jpeg]

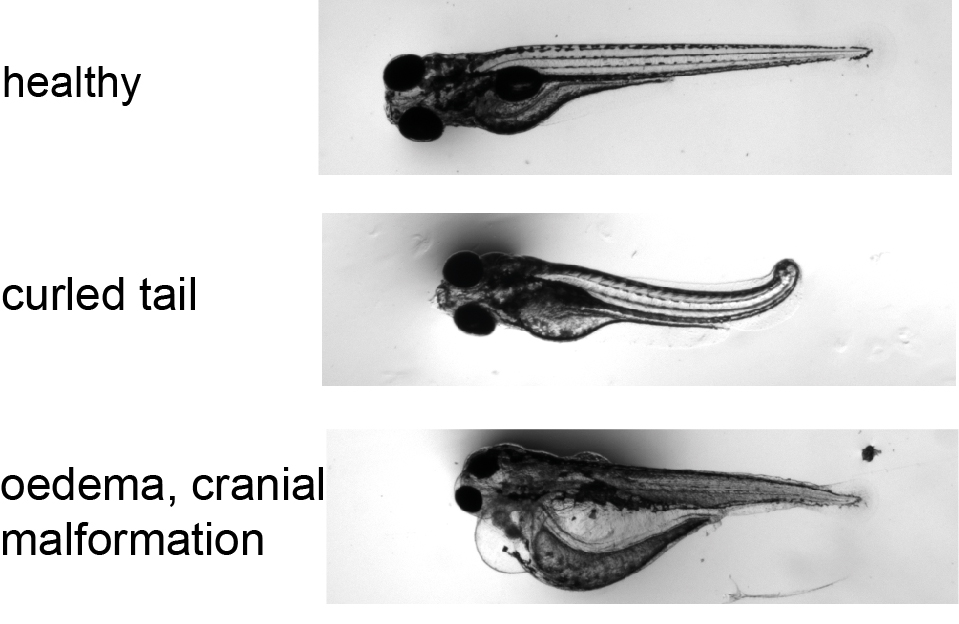

Supplement: Supplementary Figure 9 — Representative images of healthy and malformed zebrafish embryos. [file Image_9.jpeg]
